# A wearable patch for continuous analysis of thermoregulatory sweat at rest

**DOI:** 10.1038/s41467-021-22109-z

**Published:** 2021-03-23

**Authors:** Hnin Yin Yin Nyein, Mallika Bariya, Brandon Tran, Christine Heera Ahn, Brenden Janatpour Brown, Wenbo Ji, Noelle Davis, Ali Javey

**Affiliations:** 1 Department of Electrical Engineering and Computer Sciences, University of California, Berkeley, CA USA; 2 Berkeley Sensor and Actuator Center, University of California, Berkeley, CA USA; 3 Materials Sciences Division, Lawrence Berkeley National Laboratory, Berkeley, CA USA

**Keywords:** Assay systems, Diagnostic markers, Biomedical engineering, Fluidics, Characterization and analytical techniques

## Abstract

The body naturally and continuously secretes sweat for thermoregulation during sedentary and routine activities at rates that can reflect underlying health conditions, including nerve damage, autonomic and metabolic disorders, and chronic stress. However, low secretion rates and evaporation pose challenges for collecting resting thermoregulatory sweat for non-invasive analysis of body physiology. Here we present wearable patches for continuous sweat monitoring at rest, using microfluidics to combat evaporation and enable selective monitoring of secretion rate. We integrate hydrophilic fillers for rapid sweat uptake into the sensing channel, reducing required sweat accumulation time towards real-time measurement. Along with sweat rate sensors, we integrate electrochemical sensors for pH, Cl^−^, and levodopa monitoring. We demonstrate patch functionality for dynamic sweat analysis related to routine activities, stress events, hypoglycemia-induced sweating, and Parkinson’s disease. By enabling sweat analysis compatible with sedentary, routine, and daily activities, these patches enable continuous, autonomous monitoring of body physiology at rest.

## Abstract (web summary)

Low secretion rates and evaporation pose challenges for collecting resting thermoregulatory sweat for non-invasive analysis of body physiology. Here the authors present wearable microfluidics-based patches for continuous sweat monitoring at rest that enable detection of pH, Cl^−^, and levodopa for dynamic sweat analysis related to routine activities, stress events, hypoglycemia-induced sweating, and Parkinson’s disease.

## Introduction

Sweating is commonly associated with exercise and high ambient temperature, but the body naturally sweats more broadly and continuously to regulate core temperature during endogenous metabolic or stress processes, even during sedentary activities like sitting or sleeping. At-rest thermoregulatory sweat is secreted at much lower rates than during exercise—as low as a few nL min^−1^ cm^−^^2^ compared to higher exercise rates of 100’s of nL min^−1^ cm^−2^—making it challenging to collect and analyze^1^. However, near- or at-rest sweat may provide unique insight into body physiology compared to exercise, thermal, or chemically induced sweat. Specifically, the rate of resting sweat secretion can reflect sympathetic nervous system activity stemming from underlying health conditions. For example, resting sweating rate is related to defects in the central nervous system of infants^2,3^, to the severity of paresis in patients with brain infarction^4,5^, and to physiological habituation of soldiers^6^. Elevated or inhibited sweating at rest can further indicate autonomic dysfunctions, diabetes, cerebrovascular diseases, and Parkinson’s disease, as well as chronic psychological stress, anxiety, or pain^7–9^. Resting sweat is uniquely poised to give insight into these conditions by ensuring that endogenous sweating rates associated with stress, injury, or illness are not overwhelmed or confounded by the vastly higher rates associated with exercise or other external sweat triggers. In addition, low secretion rates may better preserve diffusive equilibria of biomarkers between sweat and blood, potentially making resting sweat composition more reflective of blood chemistry than other types of sweat. Finally, resting sweat is continuously generated, unlike the discrete or short-term generation of exercise or chemically induced sweat, creating opportunities for long-term monitoring of evolving body state even for impacted populations including patients or the elderly. Continuous measurement of at-rest thermoregulatory sweat rate and composition with wearable sensors can therefore be a powerful route for noninvasive health monitoring.

Accessing resting sweat remains an outstanding challenge, as low secretion rates and rapid evaporation limit the amount of biofluid volume available to be collected in a sensor for analysis. For this reason, most wearable sweat sensors have focused on exercise, thermal, or chemically stimulated sweat produced at rates of 10’s or 100’s of nL min^−1^ cm^−2^ or higher^10–23^. They are unable to draw low volumes of resting sweat rapidly into the device, limiting real-time assessment. A few platforms have targeted low nL min^−1^ cm^−2^ rates, but have failed to enable continuous analysis and, critically, accurate measurement of resting sweat rate^24,25^. Even historically, at-rest thermoregulatory sweat rate monitoring in clinical environments has required bulky instrumentation such as ventilated humidity measurement chambers, or 24-h collection periods for single-point analyte measurement^26^. Convenient, wearable devices for continuous resting sweat monitoring remain a gap in the field. This is a key outstanding challenge that must be overcome to make sweat a viable mode of health monitoring across activities, whether active or sedentary, and across user groups, whether young or old, healthy or ill.

In this work, we present a wearable patch for continuous measurement of at-rest thermoregulatory sweat composition and rate, overcoming evaporation by entrapment of sweat within a microfluidic sensing channel. Rapid uptake at low secretion rates is achieved via incorporation of a hydrophilic filler in the sweat collection well to reduce the volume of sweat that must be accumulated before it is pushed into the channel for measurement. Combining a rigid, hydrophilic insert with an overlaying hydrogel prevents sweat leakage for rapid uptake while also ensuring mechanical integrity. In addition, channel dimensions are tailored to minimize hydraulic pressure losses so that sweat gland secretory pressure is sufficient to push resting sweat into the device, while also ensuring fast movement of entrapped sweat within the channel and over embedded sweat rate sensing electrodes. These electrodes have interdigitated spokes—as the advancing sweat front meets a new spoke, a jump in admittance is detected that allows for selective flow measurement without interference from varying ionic concentration. In addition, the patch has a small footprint that allows versatile body placement even at small-area regions like the fingertips. Along with the electrical sensor for sweat rate monitoring, we integrate electrochemical sensors for pH, Cl^−^, and levodopa detection inside the microchannel for continuous analysis of resting sweat rate and compositions. These analytes are chosen to demonstrate the patch’s capabilities for ion and enzymatic sensing, and further because of the potential significance of these markers for indicating physiological state. Sweat pH could potentially reflect acid–base disorders, chloride levels are relevant to testing for cystic fibrosis and potentially for hydration status and electrolyte stores, and levodopa testing through sweat could be used toward precision medicine for Parkinson’s patients^14,27,28^. Multiplexed sensing of all three analytes is further valuable as enzymatic sensors, including the levodopa sensor, can be impacted by sample pH and ionic strength, so simultaneous tracking of these influencing parameters can be important for converting sensor signals into meaningful concentration readings. By uniting these electrochemical sensors, the presented device creates opportunities to study how the body’s endogenous sweating response relates to stress, metabolic conditions, and potentially neurological afflictions amongst other applications. We utilize the device to measure resting sweat secretion rates on various locations including shoulder, chest, bicep, wrist, abdomen, thigh, and leg, and finger. We explore dynamic sweat behaviors during light physical activities, glucose fluctuations, and control drug administration for Parkinson’s disease management. We further conduct longitudinal sweat rate monitoring over 24-h periods to detect the onset of and recovery from stress events. Our device proves to be an ideal platform to continuously or routinely monitor users’ medical conditions and physiological status during daily routines. It can also advance sweat investigations beyond what current wearable sweat sensors can provide by promoting a fundamental understanding of at-rest sweat secretion and its relation to diverse health conditions.

## Results and discussion

### Device structure

**Fig. 1:**
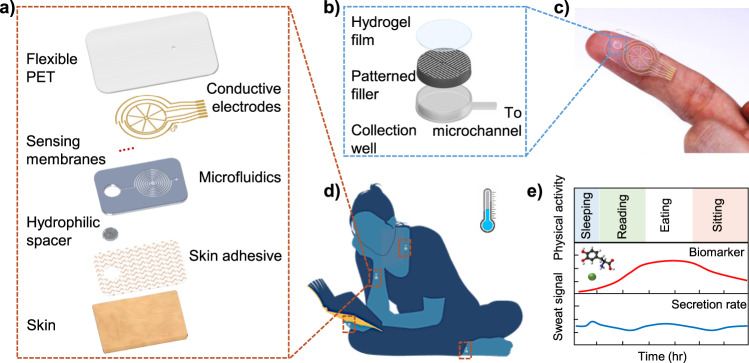
A schematic of the design, structure, and usage of the microfluidic sweat analysis patch. **a** The patch contains multiple layers. It interfaces the skin via a skin adhesive, and sweat is collected by assistance of hydrophilic filler into the microfluidics and eventually measured using sensing electrodes fabricated on a thin PET. **b** The hydrophilic filler includes a patterned SU8 mold covered with PVA film and AG-GLY hydrogel and is embedded inside the collection well. The filler enhances sweat collection by lowering sweat secretion pressure and taking up volume of the well otherwise will need to be filled. **c** An optical image of the sweat patch on a user’s finger is displayed. **d** The patch can be worn on various locations and is used to monitor sweat dynamics without interrupting routine activities. **e** It can continuously monitor both sweat secretion rate and compositions for long-term without external sweat stimulation, as schematically shown using model trends.

Our microfluidic device shown in Fig. 1 is designed to enable effective small volume collection and analysis of resting sweat. The device, includes three major components: a microfluidic layer, electrochemical and electrical sweat sensing electrodes, and a laminated hydrophilic filler. As displayed in Fig. 1a, the polydimethylsiloxane (PDMS)-based microfluidic layer contains a collection well and a microfluidic channel. The collection well interfaces the skin and its area can be modulated to acquire varying amounts of sweat. The microfluidic channel contains two intertwined spirals, and the channel connects the collection well and the outlet. The microfluidic layer is aligned and bonded together with the sweat sensing electrodes. The sensing electrodes contain four outer semicircles surrounding two interdigitated wheel-shaped electrodes. The electrochemical sensors such as pH, Cl^−^, and levodopa are functionalized on the semicircles, and the central interdigitated wheel acts as an impedance-based sweat rate sensor. Finally, the collection well is filled with a patterned SU8 filler coated with a thin saturated hydrogel layer that contacts skin for sweat uptake (Fig. 1b). The patch can be worn on areas such as the finger and wrist without interrupting human activities as pictured in Fig. 1c, d.

### Device design

Humans’ sweat secretion rates at rest vary across different body locations on average. For instance, sweat secretion rates can be lower than 10 nL min^−1^ cm^−2^ at low secretion sites such as arm and leg, and can reach on the order of 100 nL min^−1^ cm^−2^ at high secretion areas like the palm and foot^29,30^. Such secretion rates are small compared to typical sweat rates obtained by active sweat stimulation, which can be higher by an order^29,31^. To enable low resting sweat rate measurement inside the microchannel, the channel cross-section needs to be as small as possible such that temporal variations in secretion rate can be resolved by allowing fast speeds of the moving sweat front. At the same time, the channel resistance cannot be so high as to limit flow in the channel and potentially suffocate sweat gland secretion, so the channel cross section cannot be too narrow. Finally, the channel length needs to be long enough for the device to have sufficient volumetric holding capacity to enable long-term measurement on desired body locations. Here, we aim to develop a microfluidic device that can contain ~750 nL or greater such that sweat analysis can be done longer than an hour at the lowest sweat rate regions. Toward this goal, we estimated secretory pressures of the sweat gland spanning a broad range of resting sweat secretion rates from 3 to 1 µL min^−1^ cm^−2^. We established that the channel contributes to most of the device hydraulic resistance compared to the collection well. For various square cross-sectional areas and associated channel lengths that give close to 750 nL holding capacity, we calculated hydraulic pressure losses and compared these to the secretory pressure of the grand. From this, we established that a channel cross section of 70 µm × 70 µm with ~15 cm length has low enough resistance to support sweat flow across low to high secretory rates. Detailed calculations of this procedure are reported on Page 4 of the Supplementary Information. Based on these results, we chose two cross-sectional areas of the spiraling microfluidic portion for sweat rate measurement, 70 μm × 70 μm (design 1) and 200 μm × 70 μm (design 2) as depicted in Supplementary Fig. 1, with lengths shorter than 15 cm to monitor sweat rates in low and high secretion regions, respectively. Note that channels on the order of 10’s of microns wide have been previously demonstrated for wicking nanoliters of sweat off the skin surface and onto the sensor^25^. In contrast, the spiraling channel design used here is crucial not only for capturing low sweat volumes, but for efficiently drawing it over interdigitated electrode spokes for selective, continuous sweat rate measurement within a consolidated sensor footprint.

It is ideally beneficial for microfluidic collection area to be large to maximize the accessible sweat glands. However, a large collection area creates a dead volume, in which sweat firstly needs to be filled before flowing into the microchannel. This creates a lag time in sensor’s response. To address this problem, we incorporated a hydrophilic filler, containing a patterned SU8 mold and hydrogels, to occupy the dead volume and to draw sweat readily into the channel as soon as it secretes. Hydrogels have been used extensively in the wearable electronics community to create soft interfaces and to absorb and hold biofluids onto sensor surfaces, but deploying gels to enhance sweat replacement times and minimize accumulation volumes and lag times represents a key advantage in this work^32–34^. This structure overall comprises of a PVA-coated rigid SU8 component that is first inserted into the well and overlayed with an agarose–glycerol hydrogel that directly contacts skin for sweat uptake (Supplementary Fig. 2). Optical images of the filler and the assembled microfluidic are shown in Supplementary Fig. 3. We did not use hydrogel alone as a filler because it can dilute sweat compositions and hence put a challenge on the detection limit and sensitivity of electrochemical sensors, so we instead use only a thin hydrogel layer and occupy the remaining dead space with the rigid filler. The ameliorating effects of this combination on mixing and analyte dispersion are detailed in the Supplementary Information on Page 7. The filler further contributes to mechanical integrity, inhibiting collapse of the collection well under pressures which could otherwise artificially force fluid into the channel and create artefacts in measured sweat rate. The SU8 filler is patterned with grooves to alternate closed-off regions that diminish well volume with open regions roughly 100 µm-wide that allow sweat to pass through and into the device. The hydrophilic film contains two layers: a polyvinyl alcohol (PVA) and an agarose–glycerol (AG-GLY) film. The thin PVA film covers the entire SU8 filler. A single PVA layer is brittle and can easily expose the hydrophobic pathway along the cracks. This will introduce pressure against sweat secretion due to surface tension and can prevent effective transport of sweat from the skin surface into the channel. By addition of the deformable AG-GLY gel^35^ with high hydrophilicity, sweat from the collection area can be drawn into the gel and transported to the microchannel more effectively. Therefore, the AG-GLY film covers the top surface of the filler and is directly in contact with the skin. Without the hydrophilic filler, volumetric calculations show that a collection well with a 5 mm diameter and a 400 μm thickness will require more than 2 h to fill the well if sweat secretes at 300 nL min^−1^ cm^−2^ while taking over 30 min and 200 h for extreme rates of 1 µL min^−1^ cm^−2^ and 3 nL min^−1^ cm^−2^, respectively. The integration of the hydrophilic filler enhances the collection and transports fluid into the channel within a few minutes. For a 5 mm diameter collection area, the film can hold a liquid volume of nearly 200 nL in the well. For 300 nL min^−1^ cm^−2^, it takes approximately 3 min to fill the well and initiate the sweat analysis. Similarly, it takes under a minute for a rate of 1 µL min^−1^ cm^−2^ near the upper range of resting sweat secretion or around 30 min for rates toward the 3 nL min^−1^ cm^−2^ lower end when appropriately sized collection wells are used. The experimental result using a syringe pump supports this conclusion as shown in Supplementary Fig. Sa. For typical resting sweat rates ~<30 nL min^−1^ cm^−2^ ^29^, the difference in lag time is more apparent (~30 min instead of ~ a day), and sweat measurement is almost impractical for a hollow PDMS well. Note that due to its small footprint and the fact that the sensing patch is held tightly against skin via medical adhesives, the hydrogel cannot swell so much that it pushes off from the skin surface and delaminates the patch. Instead, as the hydrogel uptakes sweat, the tight seal against skin forces the hydrogel to expel this sweat into the channel. This supports rapid and leak-proof collection of resting sweat in the channel. With further investigation of the hydrophilic film and device design, it is possible to enhance the time required to initiate thermoregulatory sweat analysis at rest. Unlike prior devices which utilize hydrophilic material that has direct contact with the sensor and the skin for compositional analysis of stimulated sweat^22,25,36^, our device separates the hydrophilic filler from the sensing channel such that the sensor surface is not impacted by fluid and pressure variations in the film, and to control and fix the amount of fluid in the sensing channel for consistent sensor signals. Using the device, we also enable detection of flow rate as low as 2 nL min^−1^ as presented in Supplementary Fig. 4b.

Due to low resting sweating rates and the dimensions of the well and channel, we expect some diffusion and Taylor dispersion of analyte concentrations between when sweat is secreted on the skin surface and when it arrives at the electrochemical sensors near the entry of the channel. We perform a careful study of the time lags associated with this spread of analyte profiles in the Supplementary Information. Regions like the fingertips and hands are established to have relatively higher resting sweating rates, for which our simulations indicate a time lag of around 3 min^30^. This lag presents a limit on how updated the continuously made measurements are, but is well below the time scale over which physiological changes are expected to be manifested in sweat. At lower rates, sweat intrinsically moves more slowly through the device and takes longer to arrive at the sensos, allowing more time for dispersion effects. In contrast, because sweat rate is measured simply by the rate of fluid front movement, continuous and updated sweat rate measurements can be made with negligible time lag once sweat enters the channel.

### Device feasibility for sweat collection at rest

**Fig. 2:**
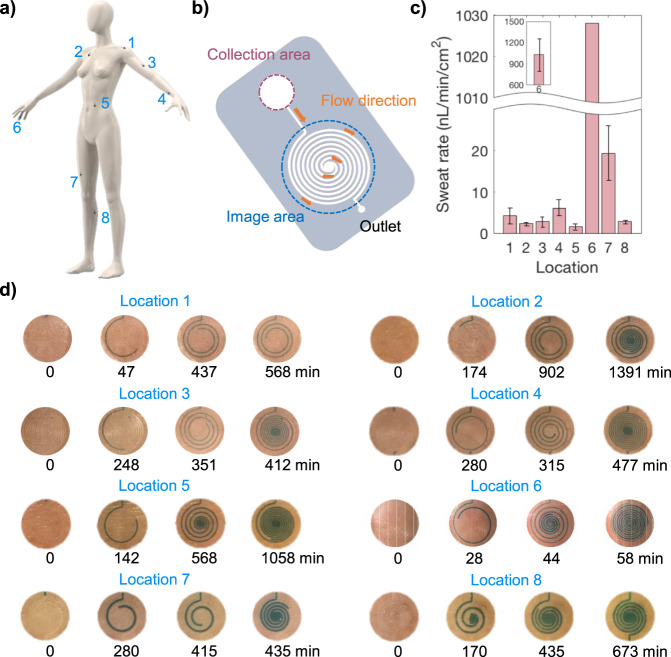
Collection of at-rest thermoregulatory sweat on various parts of the body. **a** Sweat patches were placed on 8 different locations including shoulder, chest, bicep, wrist, abdomen, finger, thigh, and calf. **b** The sweat patch used for collection and imaging is displayed. **c** The bar graph shows a subject’s local average sweat rates of regions indicated in (**a**) based on optical tracking of sweat in the microchannel from images like those in (**d**). **d** Optical images of microfluidic sweat collection at different locations and times are displayed. Note that collection areas and microfluidic dimensions are different for each location. Two different dimensions discussed in the Results section were utilized, and a collection well with diameter varied from 3 to 10 mm were used. Measured rates were normalized by the collection area.

It is important to explore at-rest thermoregulatory sweat secretion rate as it is modulated not only by environmental conditions and physical activities but also by mental stimulation and underlying health conditions^7,8,37–40^. Tracking sweat secretion routinely may help discover valuable insights into human physiology (Fig. 1e). Toward this goal, we first tested the feasibility of our microfluidic collector. We performed on-body sweat collection on various body sites, including shoulder, chest, bicep, wrist, abdomen, finger, thigh, and leg as displayed in Fig. 2a. The patches were worn by a volunteer individual (subject 1) for 24 h, and optical images were taken periodically. Patches with different collection areas were used to capture sweat rate in a practical time frame; specifically, smaller collection area was used in high secreting regions like the fingers while larger areas were used in lower secreting regions like the chest as described in the Supplementary Information Table 1. The subject was asked to refrain from moderate to vigorous physical activities during the 24-h time frame. An example of the collection area and the imaging area of the patches are displayed in Fig. 2b. Depending on the targeted regions, the patches differed in collection area and microfluidic dimensions. Color dye was used in the hydrogel to ensure sweat flow in the channel could be clearly observed. The first images in each location were taken as soon as sweat secretion began in the image area. It took 2–60 min to begin collection depending on targeted locations. Figure 2c shows a bar chart of average sweat rates on the eight locations. These values are obtained optically based on Fig. 2d, with Supplementary Fig. 5 detailing the method of calculating sweat rate from images of dyed sweat progression in the channel. Further, the patches were put on three additional subjects to measure sweat secretion rates at various locations. Measured sweat rate averages are displayed in Supplementary Table 2. According to our results, the finger has the highest secretion rate that can range between the order of 0.1 and 1 μL min^−1^ cm^−2^. All other regions show relatively low secretion rate of 1–20 nL min^−1^ cm^−2^. The results agree with the literatures which showed that palm and fingers have the highest secretion rate^29^. Majority of our measured sweat rates are slightly lower than reported rates in literatures possibly due to lower environmental temperatures and humidity used in the experiments. To demonstrate the reproducibility of the sweat rate measured by the patch, we also conducted a trial where we had a subject wearing the patches on two adjacent locations on the thigh. The data is displayed in Supplementary Fig. 6. The two patches show similar sweat rate trends and nearly identical rates when the subject was sleeping. In addition, Supplementary Fig. 7 compares sweat rate measurement from two patches placed near each other on the forearm, with one oriented horizontally and the other vertically. The orientation does not greatly impact sweat uptake into the device. Finally, note that the when the patch is worn on a finger, it can extend across the upper finger joint (as seen in Fig. 1c) and inhibit bending. While this is not highly disruptive as a relaxed finger is naturally relatively straight at the upper joint, more compliant substrates can be used in future to ensure reliable sweat uptake even with significant bending across covered joints.

There are a few factors that may induce uncertainty in measured sweat rates values. They include possible sweat migration into the collection area from other parts underneath the patch. In addition, there is a possibility of higher sweat rate in the collection area to make up for the perspiration that may be hampered in the rest part of the device. These factors can result in overestimation of the measured sweat rates; however, the relative sweat rates will not differ. To investigate the first concern, we spot colored dye on the underside of the patch. After device removal, we observe that skin is dyed just in the region of the collection well and not in surrounding regions, confirming that there is no lateral sweat leakage or transfer from the collection well, and all sweat produced in that area is forced into the device for measurement. The dyed sweat can be visually monitored as it flows in the channel to optically validate electrical sweat rate measurements or as an independent visual measurement scheme enabled by this patch. This scheme for optical sweat rate tracking is realized via discrete photographs of sweat progression within the channel as in Fig. 2.

As for the second factor that could impact sweat rate accuracy, namely compensatory sweating effects, all devices covering sweat glands can induce the same effect, and this requires careful studies in the future. Local heat generation due to on-body attachment of the patch must also be considered as it could potentially elevate sweating rates^18^, but negligible local heating is observed as demonstrated in Supplementary Fig. 19 due to the small patch size and at or near rest conditions. We compared the measured sweat rates from the patches with more traditional gravimetric analysis. For the latter, an absorbent pad is held against skin for sweat accumulation and weighed before and after each sweat collection that lasts approximately 20–30 min. The pad is placed in a shallow 0.5 cm^2^ chamber to minimize evaporation during sweat collection. A new patch was used in each sweat collection. The patch and the pad were placed on ring and pinky fingers to simultaneously collect sweat. Results are displayed in Supplementary Fig. 8, which shows that the patch collects ~2 times larger sweat amount per unit area than the pad. It is important to note that evaporation of the absorbent pad during removal from the skin surface and weighing can have significant effect on the measured amount of sweat. We discovered that the evaporation rate from the pad can be 200–400 nL min^−1^ cm^−2^, which is the same order of measured sweat rates. Hence, gravimetric measurement error can be on the order of 100%. This can lead to a lower sweat rate measured by the gravimetric method. This shows a key advantage of our device as it minimizes the uncertainty arisen from the evaporation. When dealing with low volumes and rates associated with at-rest sweat, our device encapsulates sweat immediately and uses a narrow channel to create rapid movement of the sweat front, translating into frequent and updated sweat rate measurements that overcome the evaporation and errors of gravimetric analysis. With these considerations, it is reasonable to assume that sweat under the collection area faithfully contributes to the measured sweat rate from the patch.

### Sensors characterization

**Fig. 3:**
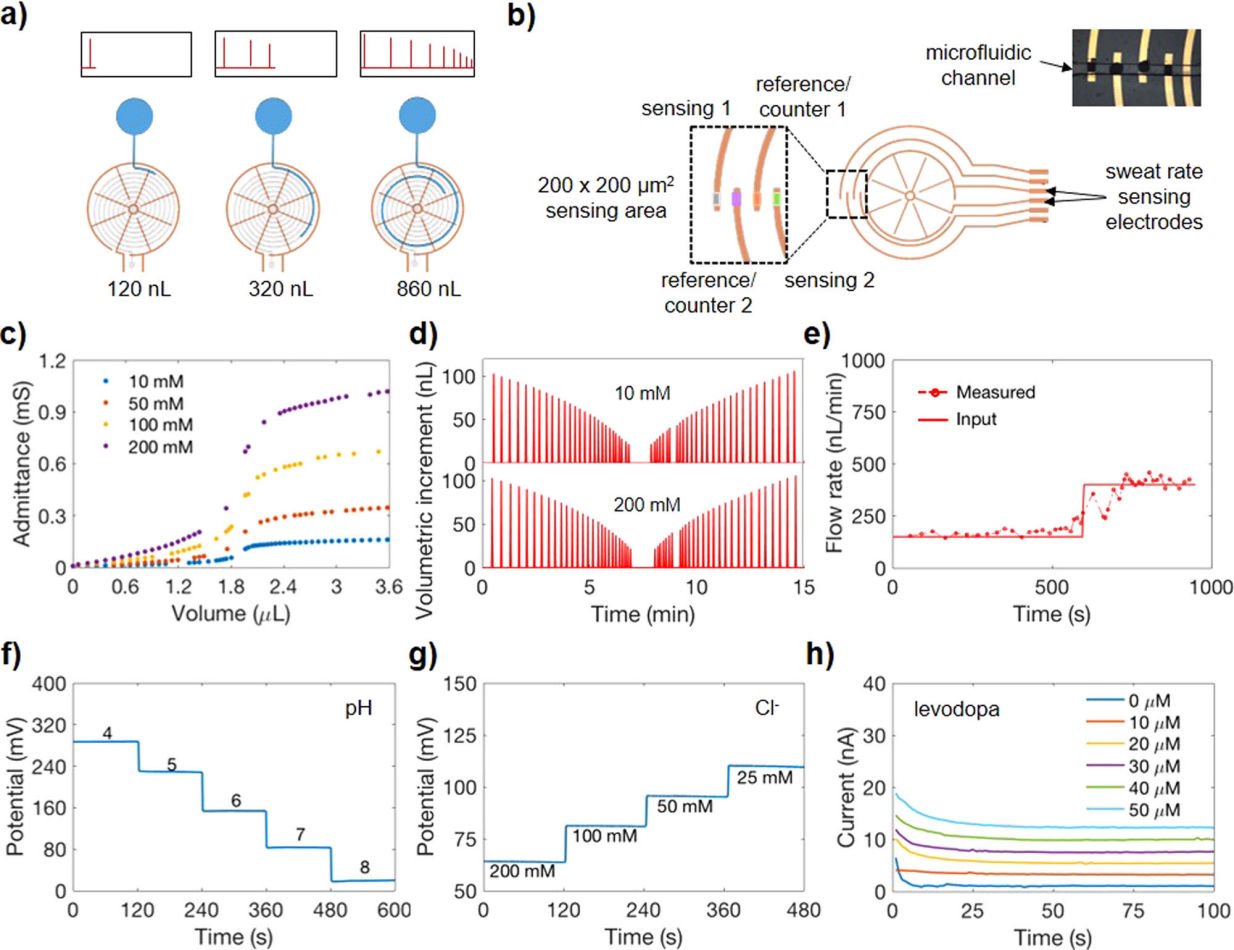
Sweat sensor characterization. **a** An impedimetric sweat rate sensing electrodes for detection of secretion rate is illustrated. An admittance (reciprocal of impedance) pulse is measured upon fluid contacting each of the radial electrode. **b** Electrochemical sensors for compositional analysis are functionalized near the tip of the four semicircles. These sensors are embedded inside the microchannel. **c** Admittance responses to solution containing NaCl concentrations of 10, 50, 100, and 200 mM. **d** Incremental volume filled inside the microchannel with respect to time is plotted when 10 and 200 mM NaCl solutions are flowed at a constant rate of 250 nL/min. The incremental volume corresponds to additional fluid filled between two adjacent radial electrodes. **e** Input flow rate from syringe pump and measured flow rate from sweat rate sensor are compared. Performance of (**f**) pH, (**g**) Cl^−^, and (**h**) levodopa sensors are presented.

In order to utilize the microfluidic patch for electrical measurement, electrical sensing electrodes are incorporated into the microfluidic. As shown in Fig. 3a, two interdigitated wheel shape electrodes are aligned with the microfluidic and act as a sweat rate sensor. The electrodes contain a total of 8–24 radial electrodes. At the initial contact, a sudden change in admittance indicates fluid entering the channel. As fluid is transported through the channel, it contacts an increasing area of the radial electrodes. With each contact by fluid, the impedance decreases because of a decrease in the resistance between the two electrodes, and a pulse indicating a change in admittance (inversely proportional to impedance) is observed. By counting the number of pulses and time interval between each pulse, the volume contained in the channel and sweat rate can be computed. In other words, as the spacing between the spokes is known and the channel cross-section is fixed, each time the sensor signal undergoes a discrete step change we can know how much additional volume of fluid was added to the channel. This allows an estimate of volumetric increment versus time, where the time points correspond to the time of the admittance step changes, as shown schematically by the signals in Fig. 3a. Note that the spacing between spokes decreases as the channel spirals inwards and increases once it starts spiraling outwards. This causes the volume increment to decrease as the fluid front moves toward the center of the spiral, and to increase as it continues to move outwards.

A larger number of radial electrodes allows for higher temporal resolution of sweat rate measurements. Electrochemical sensors located at the end of the semicircular electrodes are aligned with the microchannel as shown in Fig. 3b. This allows electrochemical analysis as soon as sweat secretes into the channel. Depending on the sensing mechanism, either electrical current or potential is monitored.

The sweat rate sensor (200 μm × 70 μm) was first characterized by measuring admittance in different concentrations of NaCl solutions at an operating frequency of 100 kHz. This frequency was chosen to minimize the capacitance contribution of the impedance and to maximize the resistive part of the impedance measurement. Figure 3c demonstrates the relationship between admittance and fluid volume in the channel for NaCl concentrations of 10, 50, 100, and 200 mM. It can be seen that at higher NaCl concentrations, the admittance between the electrodes increases due to the higher conductivity of increasing ion concentrations. In addition, increasing fluid volume in the channel gives rise to higher admittance as more ionic solution is in contact with a larger area of the electrodes, decreasing the resistance between the electrodes. To demonstrate the reliability and reproducibility of the sweat rate sensors, it is also necessary to show that the time interval between admittance pulses are the same for a given flow rate in the channel and for fluid volume between the two contacts regardless of ions concentration. Using a commercial syringe pump, 10 and 200 mM NaCl solutions were flowed at a constant rate of 250 nL min^−1^ into the sweat rate sensor. The volumetric increments between consecutive contacts is plotted as a function of time in Fig. 3d. In comparing the 10 and 200 mM plots, it can be seen that the pulses occur at the same time, indicating a reproducible calculation of sweat rate. It is also important to note that the time interval spacing between each pulse is not the same for a constant flow rate in the channel because the fluid volume between consecutive contacts decreases as fluid travels toward the center and increases as fluid travels outwards from the center toward the outlet. For a 24-electrodes with 200 μm × 70 μm channel, the time resolution is between ~4 and 20 s for 50 nL min^−1^ and can reach 2–9 min for 2 nL min^−1^. For a 24-electrodes with 70 μm × 70 μm channel design, the resolution is further enhanced. Lastly, to verify that our sweat rate device accurately returns the correct flow rate, the measured flow rate calculated from our sweat rate sensor was compared against the known input pump rate of a commercial syringe pump system. The syringe pump was used to flow 200 mM NaCl inside the microfluidic channel at an input rate of 150 and 400 nL min^−1^ as shown in Fig. 3e. It can be seen that the input pump rate is in agreement with the measured flow rate from the device, which is also evident in Supplementary Fig. 4b for lower flow rates. Note that an injection pump is used to conduct this benchtop analysis, and variation in how smoothly and consistently the pump injects at the preset rate causes fluctuations in the measured signal. This can be treated and potentially filtered as noise.

We further characterized the electrochemical sensors which have a sensing area of 200 μm by 200 μm each, given the 200 μm width of the functionalized electrode tips and the 200 μm width of the microfluidic channel in between the collection well and spiraling portion (as depicted in Supplementary Fig. 1). As shown in Fig. 3b, two electrodes serve as reference/counter electrode, and two electrodes are functionalized to detect target analytes. Detailed fabrication steps are outlined in the “Methods”. pH and Cl^−^ sensors operate by measuring the potential difference between the ion-selective electrode (ISE) and the reference electrode. The potential of the pH ISE changes with pH due to deprotonation of the ISE’s conductive polyaniline film by H^+^^27^. In contrast, the Cl^−^ ISE comprises of an Ag/AgCl electrode. A change in Cl^−^ concentration shifts the redox equilibrium between Ag and AgCl to create a measurable change in the sensor’s potential signal. Figure 3f, g shows the performance of a pH sensor in pH 4–8 McIlvaine’s buffer and a Cl^−^ sensor in solution containing 25–200 mM NaCl. Their sensitivities are measured to be 60 mV/pH and 55 mV/decade, which are close to Nernstian behavior. Figure 3h presents the performance of a levodopa sensor with a sensitivity of 0.2 nA μm^−1^, an improvement in sensitivity per area compared to our previous work^28^ due to an increased active surface area arising from modified fabrication detailed in the “Methods” section. The sensor measurement is based on current generated by enzymatic reaction between levodopa and tyrosinase. A small voltage applied to the levodopa sensor drives oxidation of levodopa by the tyrosinase enzyme, producing a Faradaic current that can be calibrated into a measure of levodopa concentration^28^. All sensors show high sensitivity within the physiological range, with linear calibration curves shown in Supplementary Fig. 9 as well as reproducibility, stability, and bending tests shown in Supplementary Fig. 10 and Supplementary Fig. 11^14,27,28^. For the levodopa sensor, decreasing the sensing area has been a challenge as signal to noise ratio becomes significant. To address this, we optimized the sensing membrane with a thin conformal layer of mediator, an enzyme-immobilized layer, and a hydrophobic micellar membrane. Our sensor shows 2.5× enhanced sensitivity per unit area compared to the previously developed sensor^28^ despite its smaller detection area, and has a response time under 20 s. Based on noise and drift, the sensor is expected to be able to discern down to 3 µM as non-negligible levodopa concentrations. We additionally performed the influence of pH and ionic strength on the levodopa sensor’s performance. Supplementary Figure 12 shows that sensitivity of levodopa sensor decreases with decreasing pH and remains relatively stable for variation of ionic strength. Selectivity of this modified levodopa sensor is shown in Supplementary Fig. 13.

To further investigate the flow effect on the sensors’ performances upon integration into the microfluidic channel, we performed flow dependence test as shown in Supplementary Fig. 14. The levodopa sensor signal shows influence from flow rate that can be understood as follows: at lower rates, levodopa concentration is mass transfer limited and changes in flow rate more significantly impact the levodopa availability at the sensor surface. Above these rates (toward 100 nL/min and beyond), mass transfer of levodopa to the sensor surface is abundant and the sensor remains at a stable, higher signal level than at lower rates. The levodopa sensor (Supplementary Figs. 8a and 15) shows an increase of approximately 0.02 nA for a change in flow rate of 10 nL min^−1^. This dependence was considered when we computed the concentration of levodopa during on-body trials. It is important to note that, for on-body levodopa sensing, the sweat rate variations were generally <30 nL min^−1^. Supplementary Figure 14b shows a pH sensor as a representative of ion sensors, and the result indicates that pH sensor is not influenced by the change in flow rate. This is likely because H^+^ ions are small; hence, they quickly dope and de-dope with the polyaniline layer without limitation on mass transport. Further, the polyaniline layer is directly accessible to target ions in solution, whereas the enzyme of the levodopa sensor is covered by protective Nafion. This causes mass transfer limitations and flow rate dependence for the levodopa sensor, but not for the pH sensor. We further conducted experiments to investigate the influence of hydrogel on capturing true concentration of injected fluid. Supplementary Figure 16a shows levodopa sensor that is initially loaded with 10 μm levodopa, and 20 μm levodopa solution was injected at a constant rate of 500 and 100 nL min^−1^. For these flow rates, the sensor took less than 3 and 15 min, respectively to start responding to a change in concentration. To eventually reach the newly injected concentration, the sensor required about 6 and 40 min, respectively. On the other hand, the pH sensor (Supplementary Fig. 16b) took about a minute to detect change in pH and 10 min to replace the detection chamber with newly injected pH for an injection rate of 100 nL min^−1^.

### Near-rest perspiration analysis during light physical activities

**Fig. 4:**
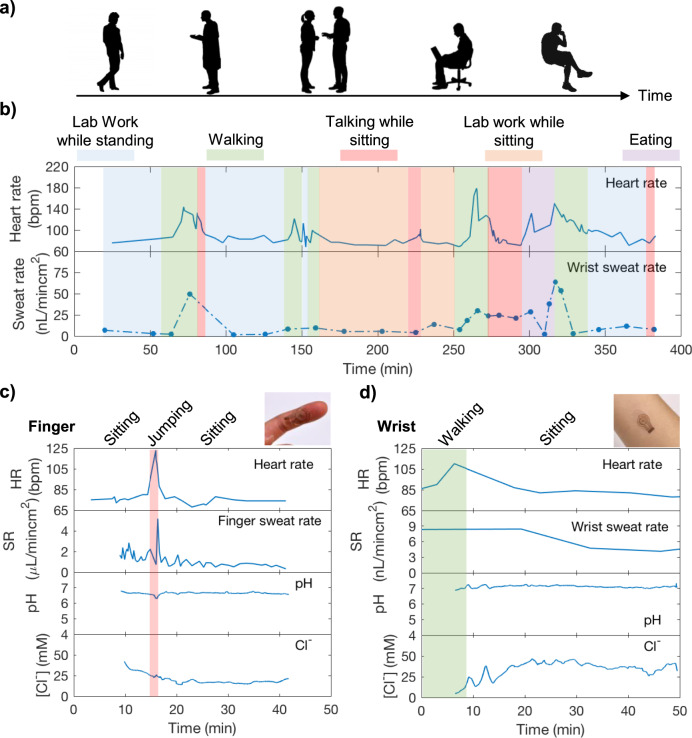
In-situ sweat analysis of a healthy volunteer while performing daily tasks. **a** The study was conducted to explore dynamic heart rate and sweat behaviors of a sedentary subject during routine activities such as talking, walking, eating, etc. **b** A subject wore the microfluidic patch and a heart rate monitor on the wrist, and heart rate and sweat rate were continuously monitored for 6 h. A subject wore the microfluidic patch on (**c**) finger and (**d**) wrist, and heart rate, sweat rate, sweat pH and Cl^−^ were simultaneously measured. Sweat measurement began 10 min and 4 h after sweat secretion began on finger and wrist, respectively.

The microfluidic patch was first used to monitor sweat dynamics to demonstrate if sweat can track different physical activities of a sedentary subject while performing routine tasks (Fig. 4a). The patch was placed on the wrist of a healthy volunteer, along with a heart rate monitor. Heart rate and sweat rate were simultaneously monitored for 6 h, with simple optical readout of sweat rate used over this extended sensing duration for convenience via the scheme detailed in Supplementary Fig. 5. Results in Fig. 4b show that wrist sweat rate generally tracks heart rate stemming from various physical activities such as taking a walk and performing lab work. Specifically, sweating rates remained relatively low along with heart rate during more sedentary periods, while intervals of walking and other activities caused both to rise and subsequently fall. We additionally conducted on-body sweat analysis on the finger and the wrist of a volunteer subject using electrical sweat rate measurement. A collection well of 3 mm diameter was used on the finger while an 8 mm diameter was used on the wrist for sweat analyses, with the larger collection area on the list accounting for the lower expected rates of secretion at this site. This allows hour-long measurement on both finger and wrist based on flow rates measured in Fig. 2c and Supplementary Table 2. To ensure microfluidic patches closely reflect actual sweat concentrations with a stable signal, we began finger and wrist sweat analyses 10 min and 4 h after sweat secreted into the sensing channel. The time scales were chosen based on the subjects’ average sweat rate shown in Supplementary Table 2 and to achieve a stable sensing signal. For instance, for an average sweat rate of 300 nL min^−1^ cm^−2^, it takes ~3 min for sweat to flow into the sensing channel. To ensure we can capture a stable signal we waited until 10 min to initiate the measurement. Figure 4c, d shows finger and wrist sweat analyses as well as heart rate measurement on a healthy subject. Similar to the previous study, sweat rate, in general, follows changes in heart rate by elevating due to periods of activity and then restoring to lower levels. Sweat pH remained stable at 6.8 and 7.1 on the finger and wrist throughout the measurement period. Sweat Cl^−^ showed slight variation initially and stabilized around 22 and 40 mM on finger and wrist, respectively. This observation is supported by the literature^41^. Finger sweat rate showed higher resolution due to faster sweat secretion rate. Resolution of wrist sweat rate can be enhanced by increasing number of radial electrodes in sweat rate sensors as discussed previously. Under our experimental conditions, we consistently observed perspiration in short time intervals (in second for the finger and in minutes for the wrist) throughout the day. Due to its ability to closely track different activities, it can be beneficial for sweat investigations associating with physical and mental stress-induced sweat.

### Sweat analysis to detect stress events over 24 h

**Fig. 5:**
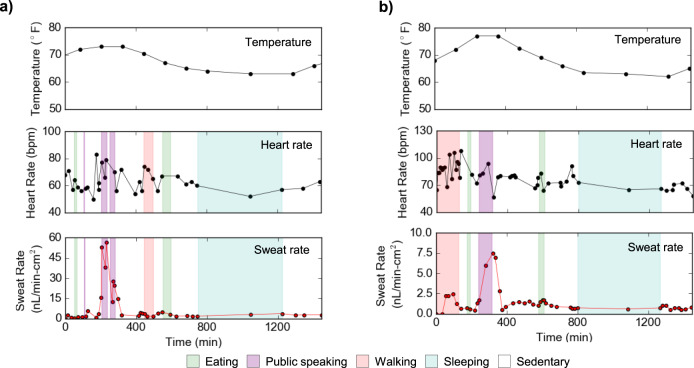
Twenty-hour in situ sweat analysis to identify stress events among routine activities. Sweat is monitored on the fingertip of a healthy volunteer along with heart rate and ambient temperature as the mostly sedentary subject performed intervals of public speaking (**a**) during a live-streamed academic conference in Trial 1, and (**b**) while teaching a class in Trial 2. The stress-inducing intervals of public speaking are associated with elevated heart rate and a sharp increase in sweat rate.

The patch was next worn on the fingertip of a healthy volunteer during two trials, 24 h each, with routine activity including eating, walking, and sleeping, while heart rate and ambient temperature were monitored simultaneously. The subject was mostly sedentary and performed intervals of public speaking including giving a presentation and answering questions in a live streamed conference in Trial 1 (Fig. 5a), and teaching a class in Trial 2 (Fig. 5b). These events generated a stress response in the body due to a combination of anticipation and public speaking that is reminiscent of the clinical standard Trier Social Stress Test^42^. Heart rate generally elevated in anticipation of and during the stress events in both trials, increasing a total of 28 bpm for the presentation in Trial 1 and 21 bpm while teaching in Trial 2. In Trial 1, baseline sweat rates during routine activities hovered around 2.8 nL min^−1^ cm^−2^ but elevated up to nearly 57 nL min^−1^ cm^−2^ during the presentation. Similarly, in Trial 2, baseline sweating rates were typically under 2.5 nL min^−1^ cm^−2^ but elevated to over 7.5 nL min^−1^ cm^−2^ while teaching. These trials demonstrate the capability of these patches to detect monitor the body’s normal sweating response during routine activities over extended and full-day time periods, and from this identify when the body moves into physiologically deviating states such as those produced during stress. Many clinical tests of stress rely on self-reported and largely qualitative measures, but this work creates potential opportunities for continuous and quantitative stress testing through resting sweat rate.

### Sweat secretion induced by metabolic changes

**Fig. 6:**
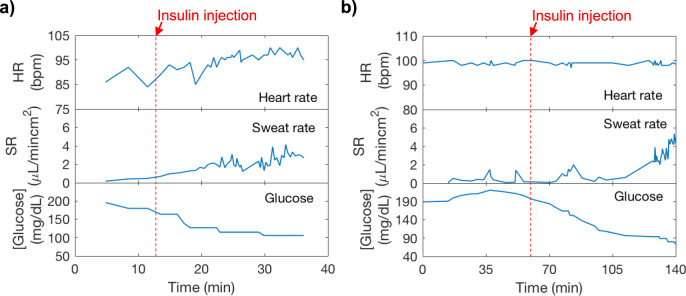
In situ sweat analysis for hypoglycemia-induced sweat analysis. Sweat secretion rate was measured along with heart rate and ISF glucose levels of a diabetic subject. Subject had insulin injection to lower glucose levels in (**a**) trial 1 and (**b**) trial 2.

The patch was further utilized to investigate hypoglycemia-induced sweat secretion. In diabetic patients, injection of insulin gives rise to hyperhidrosis due to hypoglycemia^43,44^. They can also be vulnerable to irregular heartbeat, which can be life-threatening^45^. Understanding sweating and heart complications in diabetic patients, hence, can facilitate diabetes management. Toward this aim, we performed simultaneous monitoring of heart rate, sweat rate, and interstitial fluid (ISF) glucose levels to explore heart and sweat complications during large glucose variation. A diabetic subject wore the microfluidic patch on the finger along with a pulse oximeter. The measurement was done without interrupting the routine insulin injection procedures of the diabetic patient. During the measurement duration, the subject was asked to remain sitting without vigorous movements. ISF glucose data was recorded via Dexcom G6 continuous glucose monitor. Figure 6a, b shows measurements obtained from the two trials on the diabetic subject. In both trials, glucose was initially high when the measurement began, and the sweat rate remained relatively low between 0.5 and 1 μL min^−1^ cm^−2^. After insulin was injected, glucose started to decrease rapidly. In the meantime, an increase in sweat rate was observed. When glucose further decreased lower than 90 mg/dL in Fig. 6b, there was a dramatic increase in sweat rate up to 5 μL min^−1^ cm^−2^. Heart rate remained relatively unchanged during low glucose level. Based on our results, significant decrease in glucose level is accompanied by a rise in sweat rate while no clear heart rate irregularity is observed. To develop this qualitative relation further, larger population studies must be conducted in future to quantitatively relate low glucose events and elevated sweating at rest.

### Levodopa sensing for Parkinson’s disease management

**Fig. 7:**
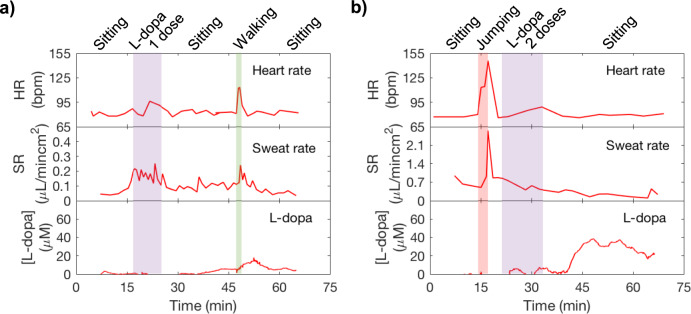
In situ sweat analysis to assist Parkinson’s disease management on daily basis. A healthy subject wore the microfluidic patch on the finger and had broad beans intake of (**a**) 1 dose = 100 g and (**b**) 2 doses = 200 g during the measurement duration. (levodopa = L-dopa).

Levodopa is a first-line drug for treating Parkinson’s disease. It has been reported that long-term intermittent oral dosage of levodopa causes fluctuation in plasma levodopa concentrations and leads to unpredictable responses such as motor fluctuations and dyskinesia; thus, continuous monitoring of levodopa is important to circumvent such unforeseen responses^46^. Sweat has been reported to contain foreign drugs, including levodopa^47,48^. Sweat is a promising noninvasive way to continuously monitor levodopa level inside the body. It may also facilitate finding an optimal dosage and interval that is personalized to each patient. In addition, Parkinson’s patients usually suffer from abnormal sweating. Hyperhidrosis occurs when the blood levodopa concentration is low^8,49^ Therefore, studying sweat behavior and monitoring levodopa concentration can assist management of Parkinson’s disease. Herein, we conducted on-body trials to study how sweat levodopa evolves within our body. A healthy subject was asked to consume 100 and 200 g intake of broad beans which contain levodopa^50^ to observe sweat levodopa relation to broad beans intake. In this study, boiled broad beans which were reported to contain approximately 0.6 wt% levodopa were used^51^. This corresponds to levodopa intake similar to that of levodopa medication consumed by Parkinson’s patients in a day. Levodopa sensors were calibrated in sweat as shown in Supplementary Fig. 17 to ensure measurement accuracy and account for batch variation in absolute sensor signal. A sweat collection well of 3 mm diameter was used. In Fig. 7a, it was observed that levodopa was detected in sweat approximately 20 min after initial intake and its concentration peaked at 35 min after intake. The peak concentration was measured to be approximately 13 μm when the subject had 1 dose of levodopa (1 dose of levodopa = 100 g of broad beans). In Fig. 7b, the subject again consumed 200 g of broad beans, and levodopa was measured approximately 20 min after initial intake. Its concentration peaked at 35 μm, 30 min after initial intake and slowly decreased. Additional trials presented in Supplementary Fig. 18a, b for 1 and 2 doses of levodopa intake showed similar results for the same subject. We observed that levodopa concentration in sweat generally increases with increasing doses. When other foods with minimal levodopa is consumed, no significant signal is observed (Supplementary Fig. 18c). This indicates that monitoring sweat levodopa may be a promising way to keep track of blood levodopa to assist medication management of Parkinson’s disease patients. However, the exact relations between sweat levodopa concentration, plasma levels, and intake dose can depend on diet, hydration, other physiological conditions that impact absorption and metabolism rates, and on sweat rate and secretion mechanisms. Larger population studies must be performed to better understand the influence of these factors.

In summary, we present a wearable device for rapid uptake of nL min^−1^ cm^−2^ rates of thermoregulatory sweat at rest, enabling near-real-time sweat rate and composition analysis at rest. This represents a crucial advancement for detecting sweat rates associated with underlying physiological conditions, as demonstrated in subject studies exploring the relation between at-rest sweating and metabolic and stress conditions. Expanding on these preliminary trials, this patch can be deployed for patients or applications where deregulated sweating is a priori known to indicate underlying health conditions, or can be used in exploratory subject studies to decode how sweating patterns relate to broader physiology. For example, hypoglycemia is known to qualitatively increase sweating rates as the body seeks to lower core temperature to conserve energy^52^. The presented patch can be used to more quantitatively study this phenomenon by simultaneously accumulating data on resting sweating rates and blood glucose levels, both for an individual over time and across a population of subjects. Personalized and universal correlations could then be built that enable resting sweat rate to serve as a noninvasive predictor of hypoglycemia. Similarly, excessive sweating is qualitatively known to indicate psychological duress, but more quantitative correlation studies can be performed between resting sweating rate and traditional, invasively obtained or discrete measures of mental state such as cortisol hormone levels^53^. Based on these correlations, at-rest sweat rate could then be used to continuously and non-invasively estimate stress, with applications in assessing and improving the welfare of infants, soldiers, and stroke patients, and more generally of individuals going about everyday activities. More generally, the presented patch can be used to study correlations between sweat rates and composition, helping to better understand analyte secretion mechanisms and guide how measured concentrations should be interpreted. By allowing these studies to be performed in a way that is compatible with daily routines, this work creates fresh opportunities for decoding how noninvasive parameters relate to deeper body health and for establishing the physiological utility of sweat sensing as a whole.

## Methods

### Materials

(3-Aminopropyl)triethoxysilane (APTES), polyvinyl butyral resin BUTVAR B-98 (PVB), aniline, sodium chloride, tyrosinase, glutaraldehyde, bovine serum albumin, thionine acetate salt, Nafion^**®**^ 117, tetrabutylammonium bromide (TBAB), sodium chloride (NaCl) were purchased from Sigma-Aldrich. Aniline was distilled prior to usage. Silver ink CI-4040 was purchased from EMS Adhesives. Polydimethylsiloxane (Sylgard 184) was purchased from Ellsworth Adhesives. Moisture resistant polyester film 0.0005″ was purchased from McMasterCarr (Los Angeles, CA).

### Sensor fabrication

Conductive Au electrodes were fabricated by standard photolithography and evaporation methods as detailed in our prior work^27^. Electrochemical depositions required for sensor functionalization were performed on PCI4G300 (Gamry Instruments, USA). pH sensor was prepared by growing Au microstructures at 0 V for 30 s to roughen the surface as demonstrated in previous works^54^, and then electrochemically depositing aniline solution (1 M HCl, 0.1 M aniline) by performing cyclic voltammetry from −0.2 to 1 V vs. Ag/AgCl at 100 mV/s for 25 cycles. Cl^−^ sensor was prepared by dropcasting silver ink and cured at 90 °C for 30 min. The electrode was subsequently treated with 0.1 M FeCl_3_ for 1 min. The reference electrode for pH and Cl^−^ sensors was prepared by dropcasting a thin layer of silver ink onto the Au electrode. After drying, a solution containing 79.1 mg PVB and 50 mg NaCl in 1 mL methanol was dropcasted (10 μL/mm^2^). Levodopa sensor was prepared by initially growing Au nanodendrites using pulsed voltage from −1 to 1 V at a signal frequency of 50 Hz, 50% duty cycle, and 1500 cycles, creating high surface area structures as imaged in our previous work^55^. Thionine acetate salt solution (0.25 mM) was deposited by applying 1 Hz signal frequency, pulsed voltage from −0.6 to 0 V, 90 % duty cycle, and 660 cycles. Next, 0.2 μL of Tyrosinase solution containing 99 μL of 1% bovine serum albumin, 1 μL of 2.5% glutaraldehyde, and 0.25 μL of 1 mg/mL tyrosinase was dropcasted and dried. The membrane was additionally coated with 0.2 μL of Nafion–TBAB solution which was prepared as reported in literature^56^. The levodopa sensors could be used after drying for an hour at room temperature. For long-term storage, levodopa sensors were kept at 4 °C. The shared reference/counter electrode for levodopa sensor was prepared by dropcasting silver ink and letting it dry before usage.

### Microfluidic device fabrication

Microfluidic was fabricated using standard photolithography process. SU8 photoresist was used to pattern microfluidics on a Si wafer. PDMS (base to curing agent ratio of 10:1) was poured onto the SU8 mold and cured at 60 °C for 4–5 h. The cured PDMS was peeled off and put under O_2_ plasma, along with the PET patterned with sensing electrodes at a power of 90 W, 0.2 mtorr for 1 min. 1% APTES solution was dropcasted on entire surface of the PET for 2 min. The PET was cleaned with DI water and quickly dry with N_2_. The PET was then bonded with PDMS and left it for at least an hour before usage. PDMS is soaked in DI water for 5 h prior to utilization to saturate PDMS^57^ such that permeation-driven flow is minimized^58^. Oversaturation can also be achieved through longer presoak time at high temperature. By presoaking, sweat-containing microfluidic channel evaporated/diffused through the PDMS at 0.01 nL min^−1^ cm^−2^ when the device was tested for 8 h at 21–23 °C and relative humidity of 39–42%.

### Hydrophilic filler fabrication

The patterned SU8 filler was prepared to a thickness of 200 μm on a flexible PET using standard procedures. The filler was carefully peeled off from the PET and put under O_2_ plasma. A solution containing 0.5% PVA in DI water was then drop-casted onto the filler (0.5 μL/mm^2^), ensuring a complete coverage on the entire filler (including side and back walls), and was quickly heated on a hotplate at 80 °C. The PVA film was approximately 10 μm in thickness. Once PVA dried, an AG-GLY film was placed on top of the filler. AG-GLY film was prepared by stirring and dissolving 2% agarose and 50% glycerol in DI water at 120 °C for 5 min. Once everything dissolved, ~3 mL of the solution was quickly poured into a 100 mm hydrophilic glass dish and waited until the solution dried to become a gel-like film. The AG-GLY solution is viscous and dries easily; hence, rapid pour on a hydrophilic dish is necessary for a thin and uniform thickness. Here the AG-GLY film was not directly drop-casted on the filler because of the difficulty to achieve a thin uniform coating on the entire filler if we directly drop-casted the solution. The AG-GLY film was saturated with deionized water before placing on the filler. The film is approximately 90–130 μm thick. The laminated filler was finally placed inside the collection well of the microfluidic patch.

### Device characterization

Sensor characterizations were performed on CHI1430 (CH Instruments, USA). The pH sensor was tested using McIlvaine’s buffer of pH 4–8, and Cl^−^ sensor was tested using NaCl solution of concentration ranging from 25 to 200 mM. The potential difference with respect to a reference electrode was measured for both sensors. Levodopa sensor was measured by applying 0.35 V with respect to a shared reference/counter silver electrode. Flow rate experiments were carried out using Harvard Apparatus PHD 2000 Syringe Pump.

### On-body sweat analysis

On-body human trials were carried out at the University of California, Berkeley in compliance with the human research protocol (CPHS 2014-08-6636 and CPHS 2015-05-7578) approved by the Berkeley Institutional Review Board (IRB). Both male and female subjects (between aged 21 and 45) were recruited from the Berkeley campus through campus flyers and verbal recruitments. Informed consents were obtained from all study subjects before enrollment in the study. The trials indicated in Figs. 2 and 4b were conducted at 20–23 °C and 39–50% relative humidity. Trials in Fig. 5 were conducted at 40–50% relative humidity with temperatures indicated in the figure. The trial in Fig. 7b was conducted at 22 °C and 43% relative humidity. All other trials were conducted at 21 °C and 40% relative humidity. Targeted locations for sweat analysis were wiped with alcohol swab and gauze before application of the microfluidic device. Subjects were allowed to wear comfortable clothing. For heart rate measurements, a pulse oximeter (Zacurate Model 500DL) was used. The double-sided adhesive that was laminated between the skin and the patch was from Adhesive Research (93551). To ensure device could stay firmly on skin for the measurement durations, an additional adhesive (93690 from Adhesive Research) was applied on top of the patch. No irritation from these adhesives or prolonged patch wear, and no adhesive delamination, were found during the extended on-body trials, consistent with the adhesives’ suitability of over 14 days of wear as stated by the manufacturer. For the on-body wrist sweat rate analysis, sweat rate sensors containing 24 radial electrodes were used. All the data presented were collected from separate measurements. Sweat composition data were collected using an electrochemical workstation CHI1430 (CH Instruments, USA). Electrical sweat rate data were collected using E4980AL precision LCR meter (Keysight Technologies). All the figures were plotted via Matlab.

### Statistical analysis

Standard deviations shown in Fig. 2c and reported in Supplementary Table 2 are calculated by considering multiple measurements of instantaneous sweat rate at each tested body location.

### Reporting summary

Further information on research design is available in the Nature Research Reporting Summary linked to this article.

## Supplementary information

Supplementary Information

Reporting Summary

## Data Availability

All relevant data supporting the findings of this study are available within the paper and its Supplementary information files or from the corresponding author upon reasonable request. A reporting summary for this Article is available as a Supplementary Information file. Source data are provided with this paper.

## Source data

Source Data

## Code availability

Custom code is available at https://github.com/m-bariya1/Resting-sweat-patch-custom-code.
