# Supplementary material for: A wearable patch for continuous analysis of thermoregulatory sweat at rest
Source: Nat Commun. 2021 Mar 23;12:1823. doi: 10.1038/s41467-021-22109-z (PMC7987967; doi:10.1038/s41467-021-22109-z)
Supplement: Supplementary file 1 — Supplementary Information [file 41467_2021_22109_MOESM1_ESM.docx]

Supplementary Information

A Wearable Patch for Continuous Analysis of Thermoregulatory Sweat at Rest

Hnin Yin Yin Nyein†^1,2,3^, Mallika Bariya†^1,2,3^, Brandon Tran^1,3^, Christine Heera Ahn^1^, Brenden Janatpour Brown^1^, Wenbo Ji^1,2,3^, Noelle Davis^1^, Ali Javey^1,2,3^

^1^Department of Electrical Engineering and Computer Sciences, University of California, Berkeley, California 94720, USA.

^2^Berkeley Sensor and Actuator Center, University of California, Berkeley, California 94720, USA.

^3^Materials Sciences Division, Lawrence Berkeley National Laboratory, Berkeley, California 94720, USA.

†These authors contributed equally.

**Correspondence should be addressed to A.J. (**email: [ajavey@berkeley.edu](mailto:ajavey@berkeley.edu)).

**Rationale for microfluidic channel dimensions**

**Impact of Taylor dispersion on sensor lag times and accuracy**

**Supplementary Figure 1**. Geometric parameters and dimensions for the microfluidic patch.

**Supplementary Figure 2.** Structure and function of the hydrogel-filler stack in the sweat collection well.

**Supplementary Figure 3.** Optical images of the microfluidic patch and filler component.

**Supplementary Figure 4.** Sweat rate sensor for detection of resting sweat rate in a short period of time with assistance of the hydrophilic filler and detection of resting sweat rate as low as 2 nL min^-1^.

**Supplementary Figure 5.** Image processing for optical sweat rate measurement.

**Supplementary Figure 6.** Sweat rate measured on the thigh of a sedentary individual using two microfluidic patches that are placed adjacently.

**Supplementary Figure 7.** Sweat rate measured on the forearm of a sedentary individual using two microfluidic patches that are placed adjacently with horizontal and vertical orientations respectively.

**Supplementary Figure 8.** Comparison of sweat rate measured by the microfluidic sweat patch and the gravimetric method.

**Supplementary Figure 9.** Calibration plots showing linear response of pH, Cl^-^, and levodopa sensors.

**Supplementary Figure 10.** Reproducibility, stability, and bending tests of pH, Cl^-^, and levodopa sensors.

**Supplementary Figure 11**. Extended bending tests of pH, chloride, and levodopa sensors.

**Supplementary Figure 12.** Influence of pH and ionic strength of the solution on a levodopa sensor.

**Supplementary Figure 13.** Levodopa sensor selectivity.

**Supplementary Figure 14.** Flow rate dependence test of levodopa and pH sensors inside the microfluidic channel.

**Supplementary Figure 15.** Method of compensating for flow rate effects on the levodopa sensor signal.

**Supplementary Figure 16.** Hydrogel influence of measured levodopa and pH inside the microfluidic channel at different flow rates.

**Supplementary Figure 17.** Levodopa sensor tested in sweat as background fluid.

**Supplementary Figure 18.** In-situ sweat analysis to assist Parkinson’s disease management.

**Supplementary Figure 19**. Heat generation analysis during on-body patch attachment.

**Supplementary Table 1.** Typical sweating rates and appropriate patch collection areas to enable sweat rate measurement over practical time scales at different body sites.

**Supplementary Table 2.** Measured average sweat rate of three subjects on different locations.

**Rationale for microfluidic channel dimensions**

The sweat gland is treated as a volumetric fluid source generating sweat at rate *Q* and exiting the sweat gland with secretory pressure *P_g_*. This sweat is forced into the device with hydraulic resistance *R_tot_* = *R_well_+R_channel_*, producing a pressure drop of Δ*P*= *R_tot_Q*. To sustain this flow in the device, *P_g_* must be larger than Δ*P* (ignoring atmospheric and Laplace pressures). *P_g_* higher than this required pressure does not change the flow rate *Q* in the device but instead means that sweat will exit the microfluidic channel with some nonzero pressure.

Hydraulic resistance of the channel is given by

*R_channel_* = 12 *µL*/[0.37**w*^4^]

where *L* = 15 cm, *w* = 70 um, and *µ* = viscosity = 9.5 * 10^-4^ Pa-s.^2^ By Darcy’s law,

*R_well_* = *µL*/*kA* = 2.17 * 10^13^ Pa-s/m^3^

where *k* = Darcy permeability for 2% agarose hydrogels of 100 µm thickness ≈ 620 nm^2^,^3^ *L* = 100 µm, and *A* = cross sectional area of 3-mm diameter well.

Secretory pressures of the sweat gland for exercise and sauna-induced sweat are around 2.5 kPa, while those of chemically induced sweat can reach upwards of 70 kPa.^1, 4^ Using the lower pressure as a conservative estimate, we can scale it down to lower resting sweat volumes (drawing on proportionalities indicated by Hoff’s law *P*=*σRT*Δ*C* since we expect the osmolality gradient to be related to secretion rate) assuming 2.5 kPa pressure corresponds conservatively to high rates of 20 nL min^-1^ gland^-1^. Then at extreme resting sweat rates of 1 nL min^-1^ cm^-2^ in a 3-mm diameter well, corresponding to 70.7 nL min^-1^ entering the device, we can compare the estimated sweat gland secretory pressure of 1.2 kPa to the hydraulic pressure drops associated with different channel geometries to arrive at optimal dimensions. We assume a square channel cross section and a channel length that overall allows us to hold around 750 nL in the channel.


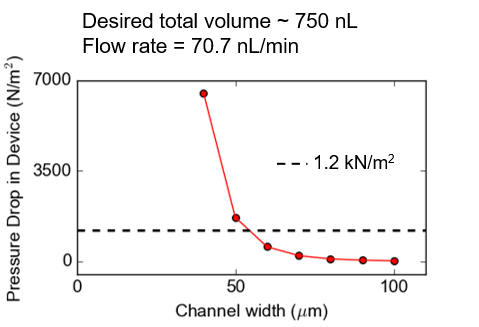


Choosing a channel width and height of 70 µm and a length around 15 cm allows a large enough volume capacity as well as a cross sectional area that is small enough to ensure fast sweat speed in the channel (necessary for high-resolution sweat rate measurement) but large enough to avoid excessive hydraulic pressure losses. In this case *R_tot_* = *R_well_+R_channel_* = 1.92 * 10^14^ + 2.17 * 10^13^ Pa-s/m^3^ = 2.1 * 10^14^ Pa-s/m^3^. Δ*P* is calculated for a broad range of resting sweat secretion and flow rates (high, medium, and low) and compared to the secretory pressure expected at those flow rates (according to *P* = 2.5 kPa * *Q*/(20 nL min^-1^ gland^-1^)) in a 3-mm diameter region with 7 glands based on typical sweat gland densities of 100 glands cm^-2^) to confirm that the gland is a sufficient pump to inject sweat into a device of these dimensions.


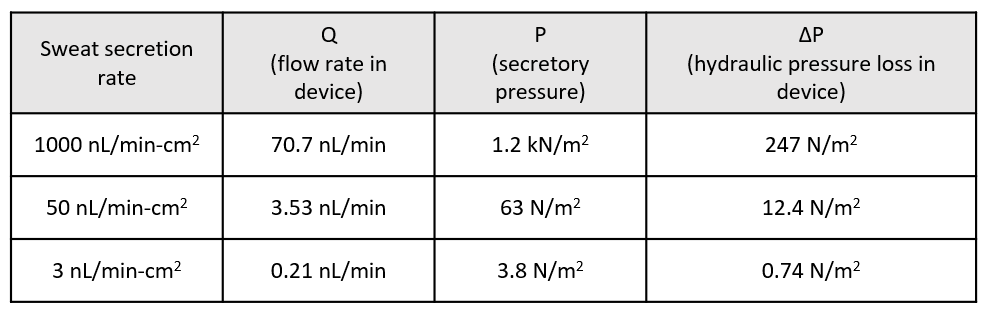


**Impact of Taylor dispersion on sensor lag times and accuracy**

Analyte diffusivity, sweat collection volume, and sweat secretion rate will impact the time lag between when sweat of a certain composition is secreted and when it is registered by the sensor. To estimate this, we consider sweat mixing and Taylor dispersion in the collection well and channel respectively using extremes of the above parameters. The following considerations are applied in our simulations:

1. The effective volume of the hydrogel-containing collection well is 72 nL for a 3 mm-diameter region. Because of the large-area proportions of the collection well, there is bulk mixing between older and fresher sweat that is treated as a continual averaging in the well.
2. We consider three sweat secretion rates (high – 1000 nL min^-1^ cm^-2^, medium – 50 nL min^-1^ cm^-2^, and low – 3 nL min^-1^ cm^-2^) that encompass a broad range of resting sweating rates. We consider sweat collection only in the 3 mm-diameter well as this broad range encompasses rates expected with the larger 8 mm opening. We consider the channel with cross section of 70 µm x 70 µm.
3. Diffusivities of H^+^, Cl^-^, and levodopa fall between 1 and 10 (x10^-9^) m^2^/s in water and in the agarose hydrogel, so these extreme values are used in the simulations.^5, 6^
4. The concentration of sweat at the sensor position depends on older sweat deeper in the channel and on sweat upstream in the channel and well. Sensor accuracy thus depends on the specific sweat composition profile, but to give a general sense of the time lags involved we consider a step concentration profile in which sweat entering the channel has concentration 0.5 for t < 0 and concentration of 1 at t ≥ 0. We solve the diffusion-advection equation in 1D (along the channel length) while incorporating Taylor dispersion to consider the temporal accuracy with which our device can reconstruct this concentration profile.

**Microchannel**: In the channel, the plots below compare smearing out of the concentration transition step at the sensor location (0.4 cm into the channel) at the three different secretion rates and extreme diffusivities. The table captures the time lag between when the step occurs at the entrance to the channel and is registered by the sensor as 90 % of the complete step in concentration. Note that the time for the sensor to register this step without diffusion or dispersion is related to the sweat secretion rate and speed in the device, so the time to 90% reconstruction must be compared to this value.

**
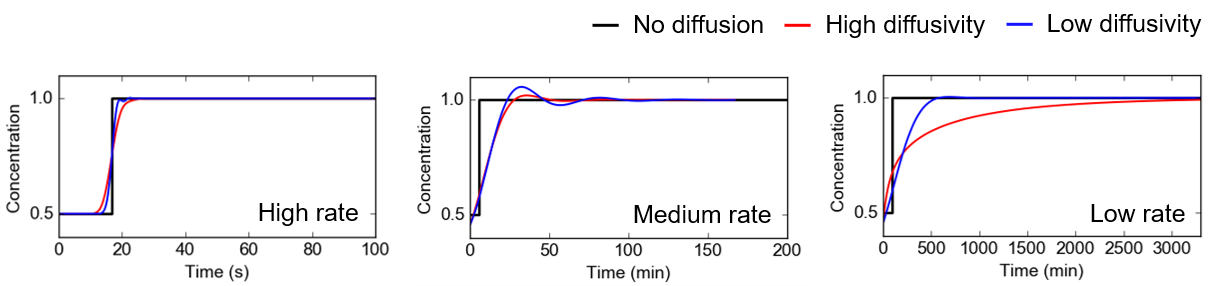
***
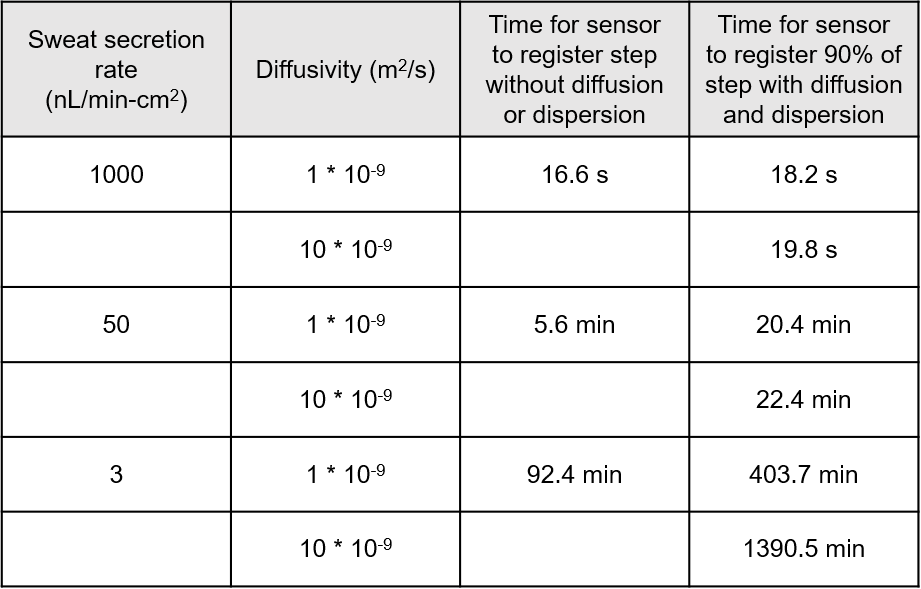
*

**Collection well:** In the collection well, the plot below averages sweat at concentration 0.5 before t = 0 with subsequent secretion of sweat at concentration 1 for t > 0 for the three sweat rates. The table below captures the the time lag between when sweat at concentration 1 starts secreting and when the well captures 90 % of the full change in concentration. Note that this time lag is related to the rate of sweat secretion and the time for adequate replacement of earlier sweat in the well.

*
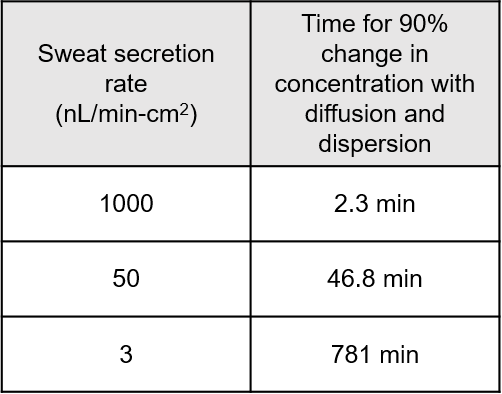
*
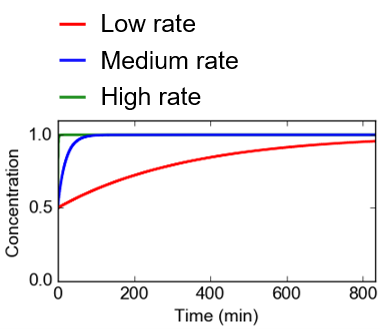


Overall, mixing and Taylor dispersion through the sections of the device indicate that at relatively high resting sweating rates on the fingertips, the sensor has a lag of around 3 minutes between when sweat at a certain composition is secreted and when it is detected at the sensor.

**Supplementary Figure 1**. Geometric parameters and dimensions for the microfluidic patch.

**Supplementary Figure 2**. Structure and function of the hydrogel-filler stack in the sweat collection well.

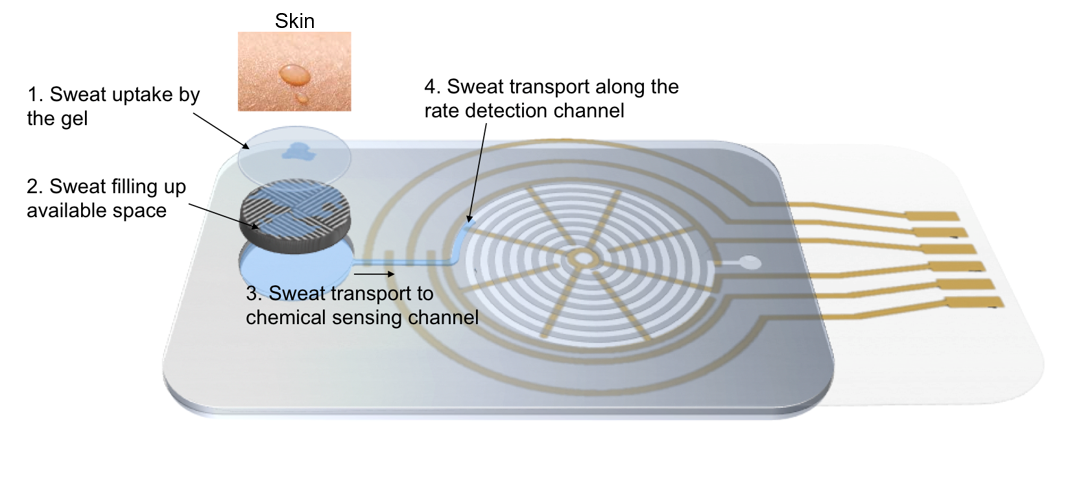


**Supplementary Figure 3.** Optical images of the microfluidic patch and filler component. a) Image of the assembled patch with PDMS microfluidic and electrode layers. b) Image of the SU8 filler after fabrication of PET backing substrate. The filler has grooves for sweat to flow through but is connected and held together due to overexposure of SU8 during lithography. The filler is peeled off the PET backing for insertion into the collection well.


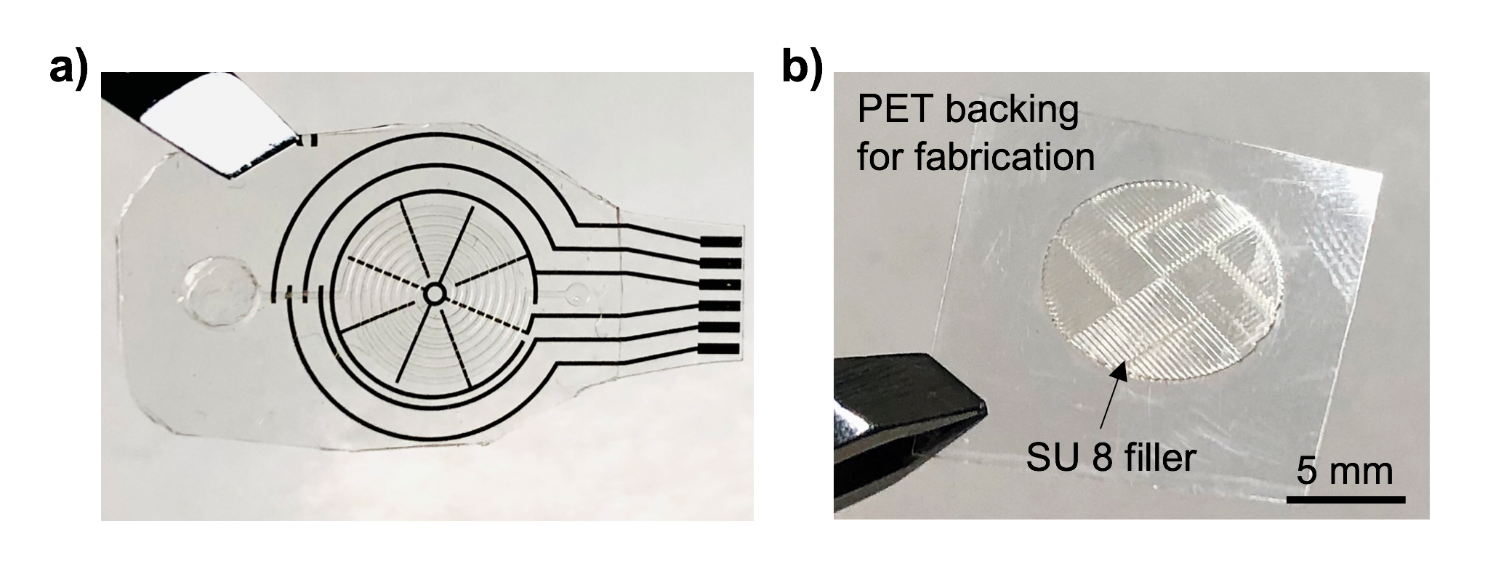


**Supplementary Figure 4.** Sweat rate sensor can be used to detect a) flow rate in a short period of time with assistance of the hydrophilic filler. The collection well of the sensor has a 3 mm diameter and can hold ~ 2.8 μL of fluid. The flow rate corresponds to 1700 nL min^-1^ cm^-2^. The sensor can detect b) flow rate as low as 2 nL min^-1^. Flow rate is measured electrically via admittance between the spoked electrodes underlying the channel. Note that injection pumps set to nL min^-1^ flow rates can generate pulsatile flow, causing some variations and transient effects in flow rate measured in the device.

**Supplementary Figure 5.** Image processing for optical sweat rate measurement. Blue dye aids in identifying the channel length occupied by sweat. In AutoCAD, a trace is made of this filled blue length and of the microfluidic spiral diameter (dashed black), which has a known length (4.8 mm for the patch worn on the finger as shown below) that can be used to scale and convert the processing software trace length into real-world units. This length is multiplied by the cross sectional area A_c_ (200 µm x 70 µm) to give the total sweat volume in the channel. This volume is subtracted from the calculated volume at the subsequent time point and divided by the intervening time to calculate the average sweat rate between when the images were recorded.


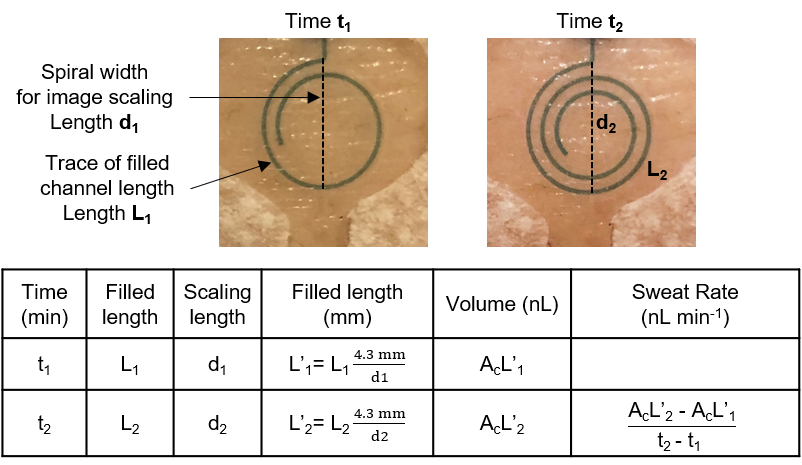


**Supplementary Figure 6.** Sweat rate measured on the thigh of a sedentary individual using two microfluidic patches that are placed adjacently.


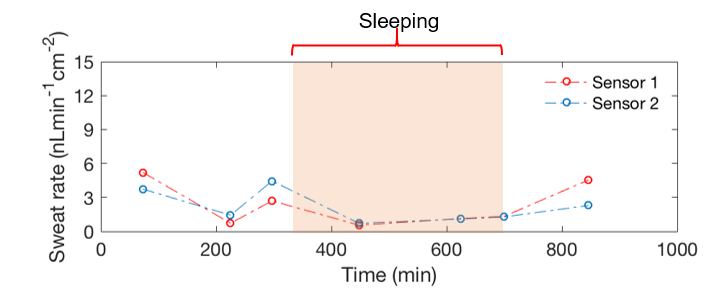


**Supplementary Figure 7.** Two microfluidic patches with 8-mm collection areas are placed near each other on the forearm, with one placed vertically and the other placed horizontally. Sweat rate is monitored for 1 hour while walking. The sensors show comparable results for the two orientations.


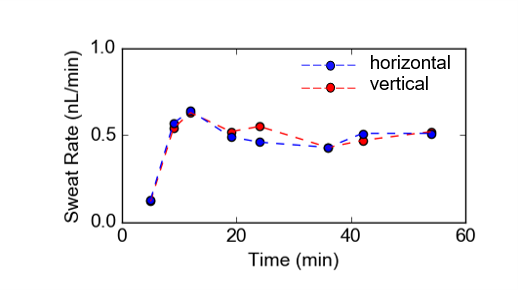


**Supplementary Figure 8.** Comparison of sweat rate measured by the microfluidic sweat patch and the gravimetric method. Sweat rate measured by the sweat patch is 2 times higher than that measured by the gravimetric method.


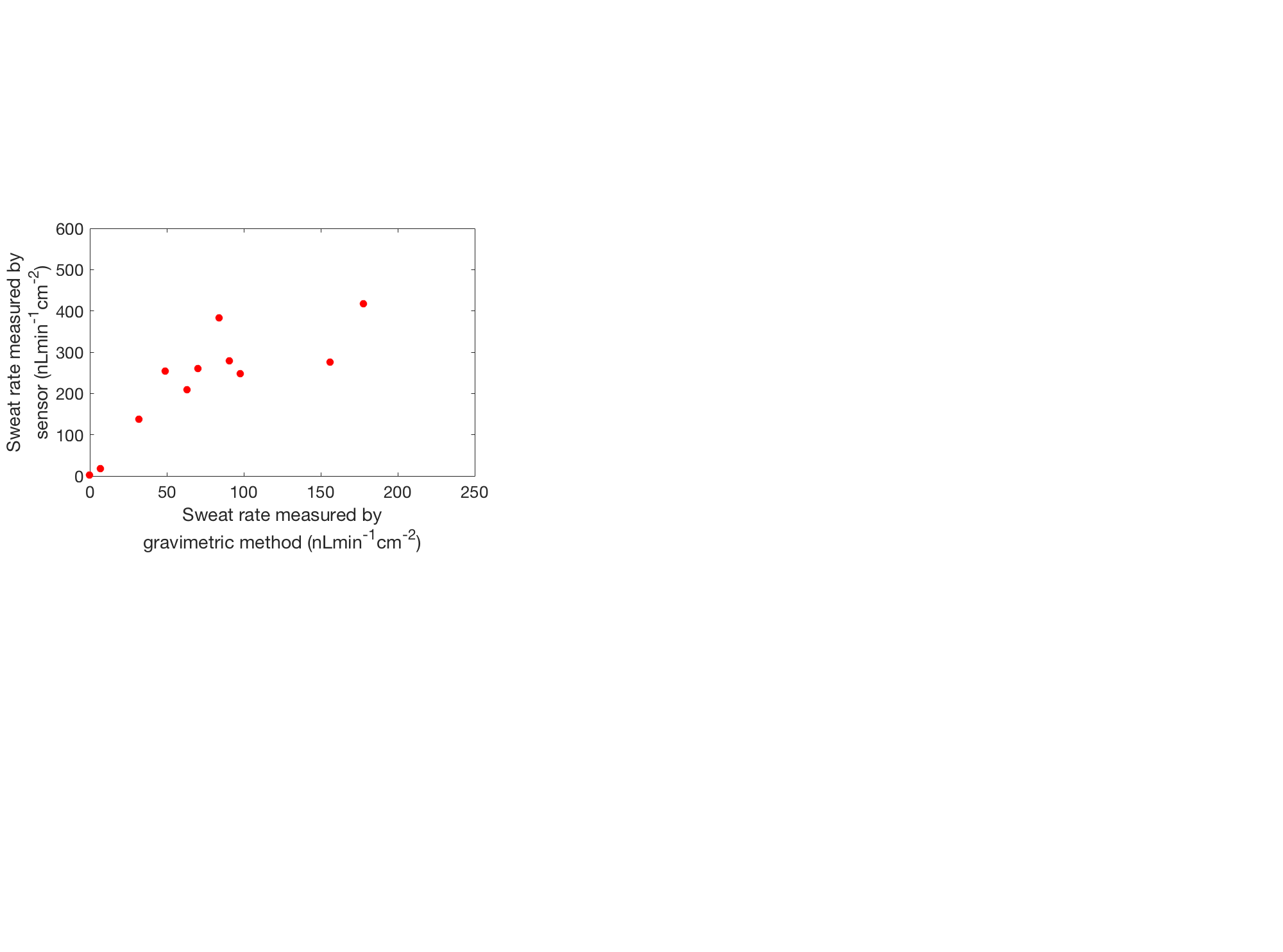


**Supplementary Figure 9.** Calibration curves of sensor signal versus analyte concentration for a) pH, b) Cl^-^, and c) levodopa. Data is obtained from the potentiometry and chronoamperometry curves in Figure 3f-h and demonstrates each sensor’s linear response.


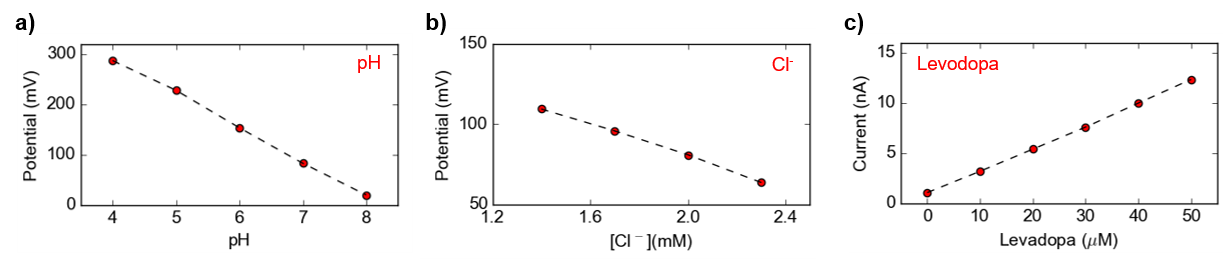


**Supplementary Figure 10.** (a)-(c) Reproducibility, (d)-(f) stability and drift analysis, and (g)-(i) bending tests using 0.66 cm radius of curvature for the pH, Cl^-^, and levodopa sensors.


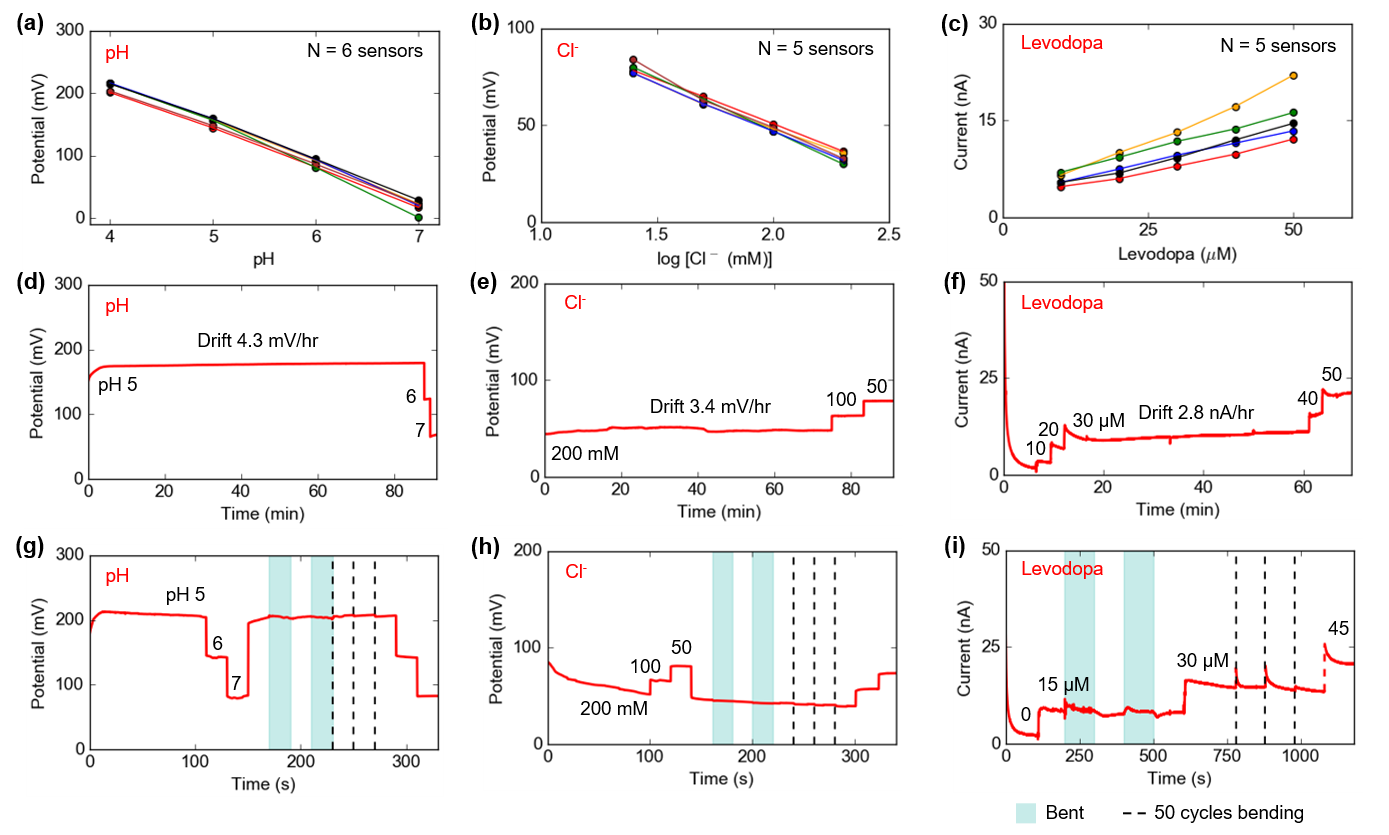


**Supplementary Figure 11.** Extended bending tests of pH, chloride, and levodopa sensors. Sensor signals are reported before bending and after 200, 400, and 800 cycles of bending with 0.66 cm radius of curvature.


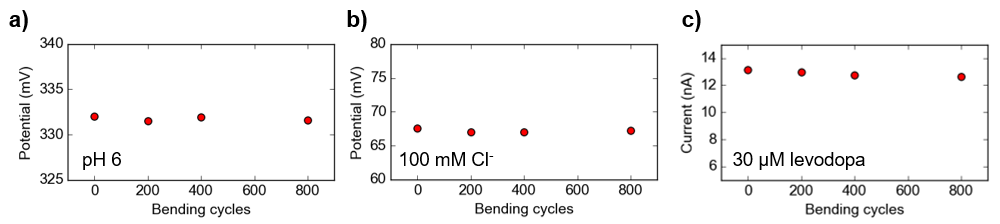


**Supplementary Figure 12.** Influence of a) pH and b) ionic strength of the solution on n=3 levodopa sensors. Note that a decrease in the sensitivity of 0.5x PBS is due to a slight decrease in pH of the buffer solution.


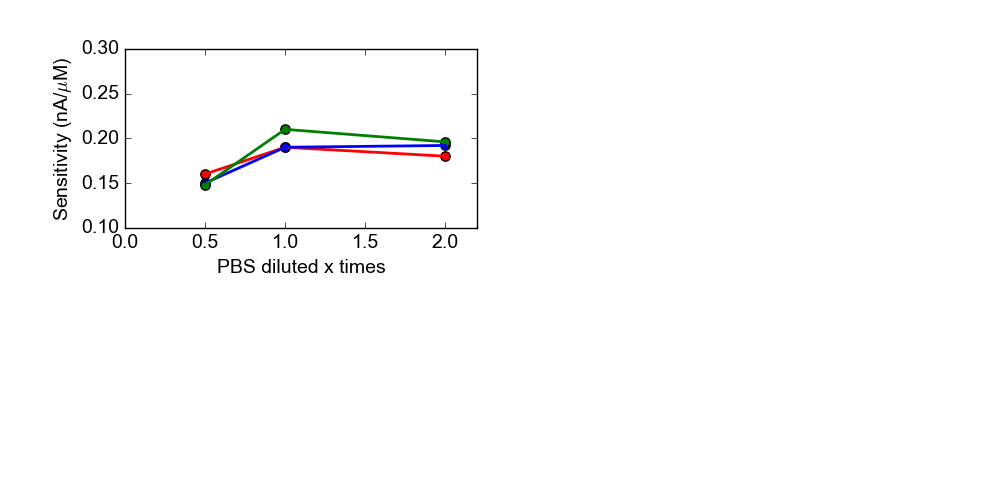

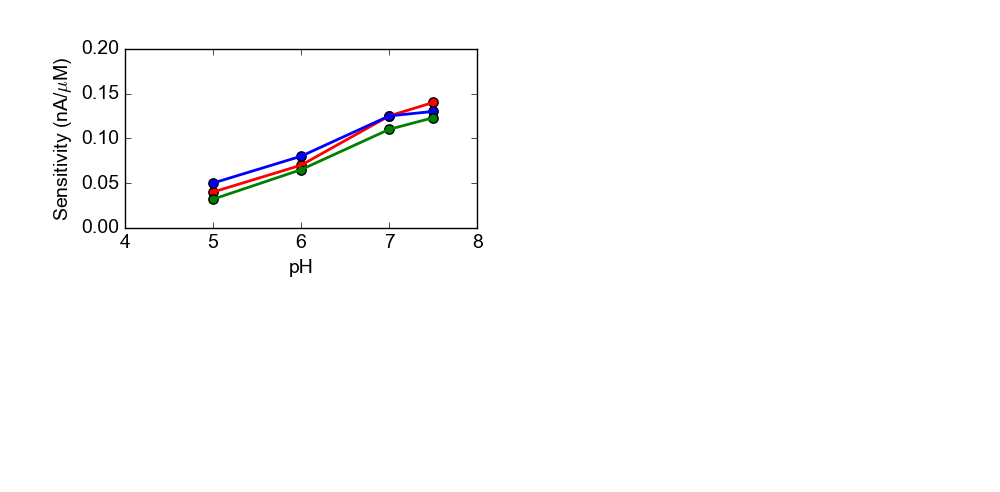


**a)**

**b)**

**Supplementary Figure 13.** Levodopa sensor selectivity against common interferents in sweat, including uric acid, ascorbic acid, and glucose.


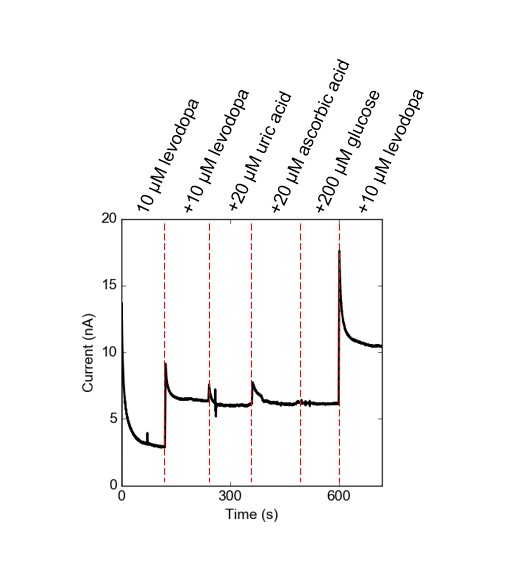


**Supplementary Figure 14.** Flow rate dependence test of a) Levodopa and b) pH sensors inside the microfluidic channel. Displayed numbers in the plot indicate flow rates in nL min^-1^.


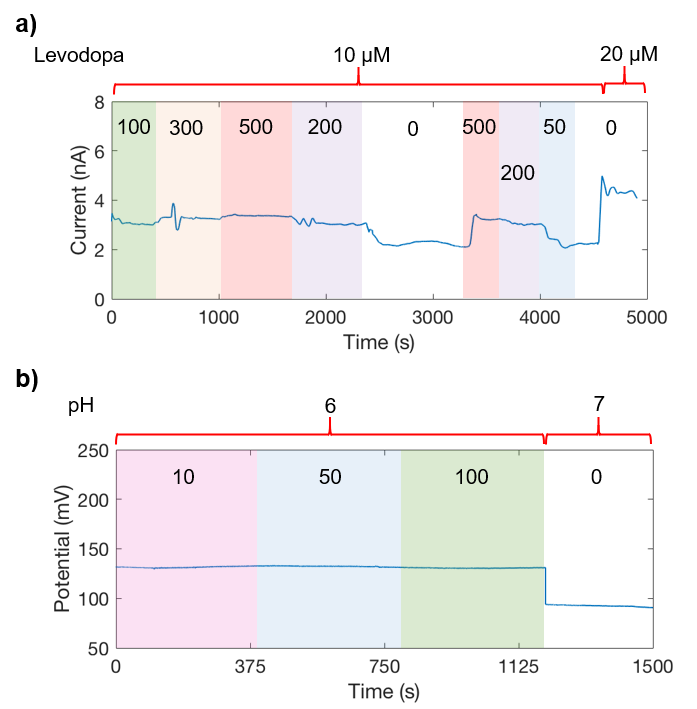


**Supplementary Figure 15.** Method of compensating for flow rate effects on the levodopa sensor signal. Off-body calibration of the levodopa sensor with no flow before on-body use is used to establish the sensor baseline. Using the on-body sweat rate measurement, the on-body levodopa signal can then be corrected using the flow rate dependence shown in a) (derived from Figure S6 (a) above) to produce the compensated curve shown in b).


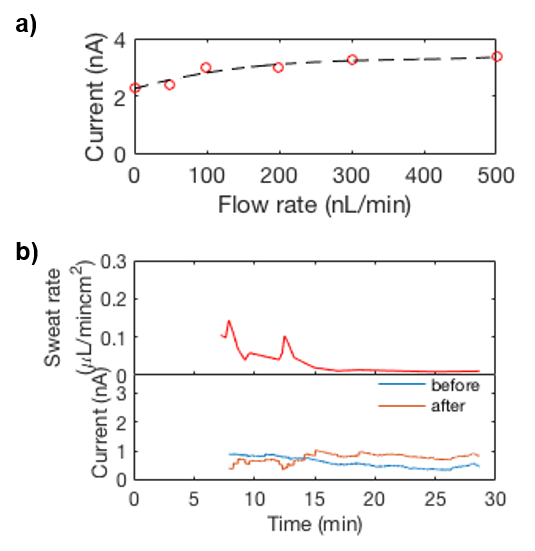


**Supplementary Figure 16.** Hydrogel influence of measured a) Levodopa and b) pH inside the microfluidic channel at a constant flow rate. Note that potential overshoot for the pH sensor is due to ion-exchange and temporary, local concentration differences as the membrane equilibrates with new solution. Further, upwards drift of the levodopa sensor upon changing the sample concentration arises due to equilibration as the higher concentration reaches and stabilizes at the sensor’s enzyme layer.


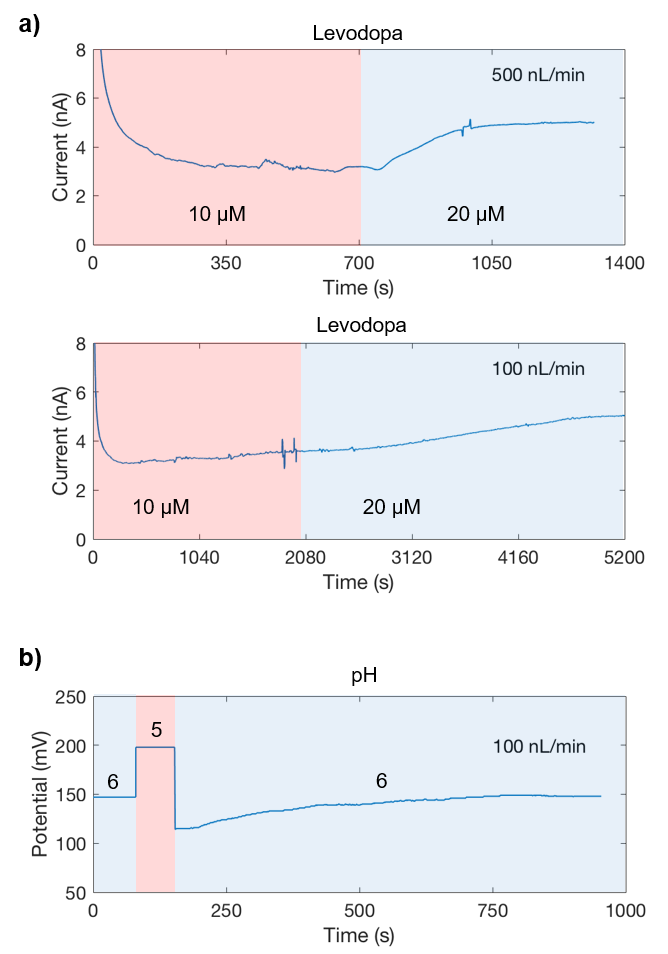


**Supplementary Figure 17.** Levodopa sensor tested in sweat as background fluid.


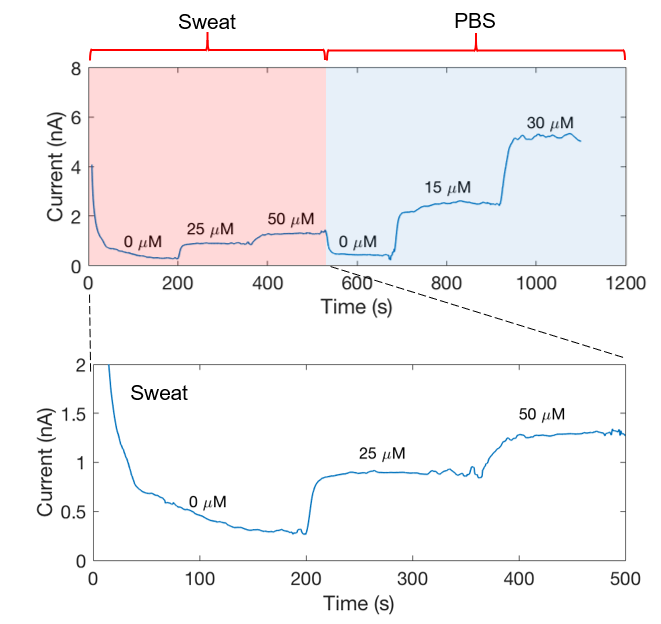


**Supplementary Figure 18.** In-situ sweat analysis to assist Parkinson’s disease management. A healthy subject wore the microfluidic patch and had broad bean intake of a) 1 dose = 100 g and b) 2 doses = 200 g during the measurement duration. c) The subject had spinach during the measurement duration as a control trial. Note that a patch containing 8 radial, interdigitated electrode spokes for sweat rate measurement was used in the trials in a) and c), while a patch with 24 radial electrode spokes was used in b).


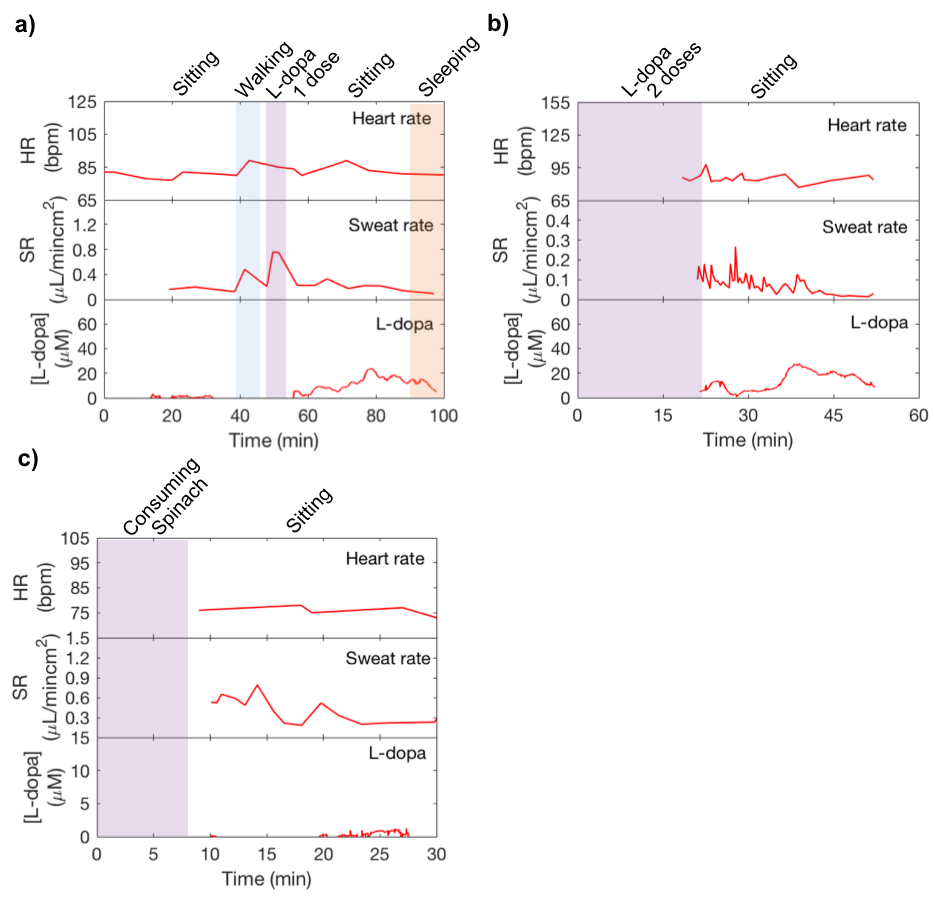


**Supplementary Figure 19.** Heat generation analysis during on-body patch attachment. Thermal infrared images captured 5 min and 90 min into on-body sensor wear. There is negligible difference in local skin surface temperature produced by the patch.


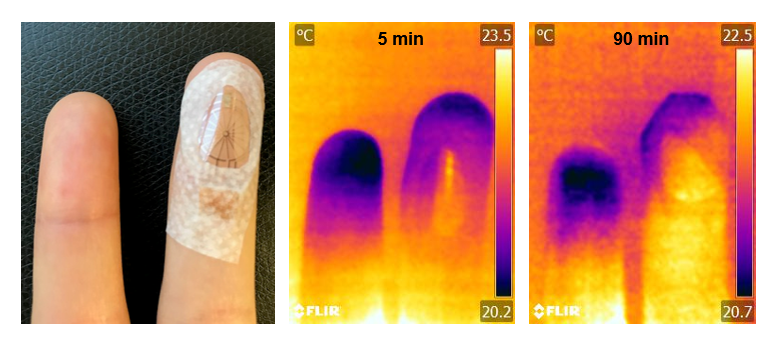


**Supplementary Table 1.** Typical sweating rates and appropriate patch collection areas to enable sweat rate measurement over practical time scales at different body sites.

| Location | Typical sweat rate (nL min^-1^ cm^-2^) ^29^ | Collection diameter (mm) |
| --- | --- | --- |
| Chest | 10 – 40 | 10 |
| Upper arm | 10 – 150 | 5, 10 |
| Forearm | 10 – 30 | 10 |
| Abdomen | 10 – 40 | 10 |
| Finger | 60 – 200 | 3, 5 |
| Leg | 10 – 40 | 10 |

**Supplementary Table 2.** Measured average sweat rate of three subjects on different locations. Three patches are placed per subject, with “-” indicating a bare site without a sensor attached. Displayed sweat rates are in unit of nL min^-1^ cm^-2^.

| Location | Subject 2 | Subject 3 | Subject 4 |
| --- | --- | --- | --- |
| Bicep | - | 6 ± 3.3 | 9 ± 1.2 |
| Wrist | 5.5 ± 1.3 | - | 7.4 ± 3.7 |
| Finger | 300 ± 93.5 | 101 ± 34 | 620 ± 202 |
| Leg | 3.5 ± 1.4 | 2.4 ± 1.2 | - |

**REFERENCES**

1. Z. Sonner, *et al.* The microfluidics of the eccrine sweat gland, including biomarker partitioning, transport, and biosensing implications. *Biomicrofluidics* **9** (3), 031301 (2015).

2. Ojuroye O, Torah R, Beeby S. Modified PDMS packaging of sensory e-textile circuit microsystems for improved robustness with washing. Microsyst Technol [Internet]. 2019 May 18 [cited 2020 Oct 29]; Available from: https://doi.org/10.1007/s00542-019-04455-7

3. E. M. Johnson, W. M. Deen. Hydraulic permeability of agarose gels. *AlChE Journal* **42** (5), 1220-1224 (1996).

4. J. Choi, *et al.* Soft, skin-mounted microfluidic systems for measuring secretory fluidic pressures generated at the surface of the skin by eccrine sweat glands. *Lab on a Chip* **17** (15), 2572-2580 (2017).

1. M. Safaei, *et al.* Electrochemical Sensing of Levodopa in Presence of Tryptophan Using Modified Graphite Screen Printed Electrode with Magnetic Core-Shell Fe 3 O 4@ SiO 2/GR Nanocomposite. *Surface Engineering and Applied Electrochemistry* **56**, 184-191 (2020).
2. G. Schuszter, *et al.* Determination of the diffusion coefficient of hydrogen ion in hydrogels. *Phys. Chem.* **19** (19), 12136-12143 (2017).
