# Supplementary material for: A wearable patch for continuous analysis of thermoregulatory sweat at rest
Source: Nat Commun. 2021 Mar 23;12:1823. doi: 10.1038/s41467-021-22109-z (PMC7987967; doi:10.1038/s41467-021-22109-z)
Supplement: Supplementary file 2 — Reporting Summary [file 41467_2021_22109_MOESM2_ESM.pdf]

nature research

Corresponding author(s): Ali Javey

Last updated by author(s): Jan 24, 2021

Reporting Summary

Nature Research wishes to improve the reproducibility of the work that we publish. This form provides structure for consistency and transparency in reporting. For further information on Nature Research policies, see our Editorial Policies and the Editorial Policy Checklist.

Statistics

For all statistical analyses, confirm that the following items are present in the figure legend, table legend, main text, or Methods section.

n/a	Confirmed	
☐	☒	The exact sample size (n) for each experimental group/condition, given as a discrete number and unit of measurement
☐	☒	A statement on whether measurements were taken from distinct samples or whether the same sample was measured repeatedly
☒	☐	The statistical test(s) used AND whether they are one- or two-sided<br>*Only common tests should be described solely by name; describe more complex techniques in the Methods section.*
☒	☐	A description of all covariates tested
☒	☐	A description of any assumptions or corrections, such as tests of normality and adjustment for multiple comparisons
☒	☐	A full description of the statistical parameters including central tendency (e.g. means) or other basic estimates (e.g. regression coefficient) AND variation (e.g. standard deviation) or associated estimates of uncertainty (e.g. confidence intervals)
☒	☐	For null hypothesis testing, the test statistic (e.g. F, t, r) with confidence intervals, effect sizes, degrees of freedom and P value noted<br>*Give P values as exact values whenever suitable.*
☒	☐	For Bayesian analysis, information on the choice of priors and Markov chain Monte Carlo settings
☒	☐	For hierarchical and complex designs, identification of the appropriate level for tests and full reporting of outcomes
☒	☐	Estimates of effect sizes (e.g. Cohen's d, Pearson's r), indicating how they were calculated

Our web collection on statistics for biologists contains articles on many of the points above.

Software and code

Policy information about availability of computer code

Data collection	No software was used.
Data analysis	Data was analyzed using Excel (2012), Matlab (R2019a), Python (2.7), and AutoCAD (2021). As mentioned in the manuscript under 'Code Availability', custom code is available at https://doi.org/10.6078/D17M6X.

For manuscripts utilizing custom algorithms or software that are central to the research but not yet described in published literature, software must be made available to editors and reviewers. We strongly encourage code deposition in a community repository (e.g. GitHub). See the Nature Research guidelines for submitting code & software for further information.

Data

Policy information about availability of data

All manuscripts must include a data availability statement. This statement should provide the following information, where applicable:

- Accession codes, unique identifiers, or web links for publicly available datasets
- A list of figures that have associated raw data
- A description of any restrictions on data availability

The authors declare that all the data supporting the findings of this study are available within the paper and its supplementary information files. Source data is available at https://doi.org/10.6078/D17M6X or available upon request from the authors.

Field-specific reporting

Please select the one below that is the best fit for your research. If you are not sure, read the appropriate sections before making your selection.

☒ Life sciences ☐ Behavioural & social sciences ☐ Ecological, evolutionary & environmental sciences

For a reference copy of the document with all sections, see nature.com/documents/nr-reporting-summary-flat.pdf

Life sciences study design

All studies must disclose on these points even when the disclosure is negative.

Sample size	Validation of microfluidic design for sweat collection and rate measurements were done on 7 participants (with data shown in Figure 2, 4 and Table S2). No sample size calculation was performed - this sample size was chosen as a small-scale population study to ensure the sweat patch effectively captured sweat across multiple subjects and trials. As the sweat patch performed reliably across all participants, no further subjects were used in this proof-of-concept work.<br><br>All other reported trials focusing on longitudinal sweating behavior were done on one participant each to consider how changes in sweat parameters relate to changes in underlying physiological states, and were repeated on the same subject (as shown in the Figures) to ensure patterns are reproducible and genuine. This was sufficient to identify qualitative trends in this proof-of-concept work. Larger subject trials with more participants will be used in follow-up work where quantitative relations between body state and sweat parameters are sought.
Data exclusions	No data was excluded from the trials or analyses.
Replication	At least two trials were presented for each study in Figures 3-7, giving consistent qualitative trends. Comparison of relative sweating rates across body sites is shown for 1 subject in Figure 2 and repeated for 3 other subjects in Table S2. Apart from this repetition for the physiological studies, sensor performance testing (calibration, drift analysis, bending tests) are repeated for 5-6 sensors in Figure S10.
Randomization	The studies shown in this work consider how sweat behavior changes over time for individual subjects during routine activities rather than placing a group of subjects in different test and control categories. For this reason, randomization was not applicable.
Blinding	Because the study did not require individuals to be separated into different control and test categories, blinding was not applicable.

Reporting for specific materials, systems and methods

We require information from authors about some types of materials, experimental systems and methods used in many studies. Here, indicate whether each material, system or method listed is relevant to your study. If you are not sure if a list item applies to your research, read the appropriate section before selecting a response.

Materials & experimental systems

n/a	Involved in the study	
☒	☐	Antibodies
☒	☐	Eukaryotic cell lines
☒	☐	Palaeontology and archaeology
☒	☐	Animals and other organisms
☐	☒	Human research participants
☒	☐	Clinical data
☒	☐	Dual use research of concern

Methods

n/a	Involved in the study	
☒	☐	ChIP-seq
☒	☐	Flow cytometry
☒	☐	MRI-based neuroimaging

Human research participants

Policy information about studies involving human research participants

Population characteristics	Male and female subjects (between aged 21 to 45) participated in this study.
Recruitment	Subjects were recruited from the Berkeley campus through campus flyers and verbal recruitments. The first set of individuals willing to participate and not having any of the exclusion criteria detailed in the protocol were chosen without bias.
Ethics oversight	This study was performed according to human research protocol (CPHS 2014-08-6636 and CPHS 2015-05-7578) approved by the Berkeley Institutional Review Board (IRB).

Note that full information on the approval of the study protocol must also be provided in the manuscript.
